# Development of virus-induced genome editing methods in Solanaceous crops

**DOI:** 10.1093/hr/uhad233

**Published:** 2023-11-17

**Authors:** Seo-Young Lee, Bomi Kang, Jelli Venkatesh, Joung-Ho Lee, Seyoung Lee, Jung-Min Kim, Seungki Back, Jin-Kyung Kwon, Byoung-Cheorl Kang

**Affiliations:** Department of Agriculture, Forestry and Bioresources, Research Institute of Agriculture and Life Sciences, Plant Genomics Breeding Institute, College of Agriculture and Life Sciences, Seoul National University, Seoul 08826, Republic of Korea; Interdisciplinary Program in Agricultural Biotechnology, College of Agriculture and Life Sciences, Seoul National University, Seoul 08826, Republic of Korea; Department of Agriculture, Forestry and Bioresources, Research Institute of Agriculture and Life Sciences, Plant Genomics Breeding Institute, College of Agriculture and Life Sciences, Seoul National University, Seoul 08826, Republic of Korea; Department of Agriculture, Forestry and Bioresources, Research Institute of Agriculture and Life Sciences, Plant Genomics Breeding Institute, College of Agriculture and Life Sciences, Seoul National University, Seoul 08826, Republic of Korea; Department of Agriculture, Forestry and Bioresources, Research Institute of Agriculture and Life Sciences, Plant Genomics Breeding Institute, College of Agriculture and Life Sciences, Seoul National University, Seoul 08826, Republic of Korea; Interdisciplinary Program in Agricultural Biotechnology, College of Agriculture and Life Sciences, Seoul National University, Seoul 08826, Republic of Korea; Department of Agriculture, Forestry and Bioresources, Research Institute of Agriculture and Life Sciences, Plant Genomics Breeding Institute, College of Agriculture and Life Sciences, Seoul National University, Seoul 08826, Republic of Korea; Department of Agriculture, Forestry and Bioresources, Research Institute of Agriculture and Life Sciences, Plant Genomics Breeding Institute, College of Agriculture and Life Sciences, Seoul National University, Seoul 08826, Republic of Korea; Department of Agriculture, Forestry and Bioresources, Research Institute of Agriculture and Life Sciences, Plant Genomics Breeding Institute, College of Agriculture and Life Sciences, Seoul National University, Seoul 08826, Republic of Korea; Interdisciplinary Program in Agricultural Biotechnology, College of Agriculture and Life Sciences, Seoul National University, Seoul 08826, Republic of Korea

## Abstract

Genome editing (GE) using CRISPR/Cas systems has revolutionized plant mutagenesis. However, conventional transgene-mediated GE methods have limitations due to the time-consuming generation of stable transgenic lines expressing the Cas9/single guide RNA (sgRNA) module through tissue cultures. Virus-induced genome editing (VIGE) systems have been successfully employed in model plants, such as *Arabidopsis thaliana* and *Nicotiana* spp*.* In this study, we developed two VIGE methods for Solanaceous plants. First, we used the *tobacco rattle virus* (TRV) vector to deliver sgRNAs into a transgenic tomato (*Solanum lycopersicum*) line of cultivar Micro-Tom expressing *Cas9*. Second, we devised a transgene-free GE method based on a *potato virus X* (PVX) vector to deliver *Cas9* and sgRNAs. We designed and cloned sgRNAs targeting *Phytoene desaturase* in the VIGE vectors and determined optimal conditions for VIGE. We evaluated VIGE efficiency through deep sequencing of the target gene after viral vector inoculation, detecting 40.3% and 36.5% mutation rates for TRV- and PVX-mediated GE, respectively. To improve editing efficiency, we applied a 37°C heat treatment, which increased the editing efficiency by 33% to 46% and 56% to 76% for TRV- and PVX-mediated VIGE, respectively. To obtain edited plants, we subjected inoculated cotyledons to tissue culture, yielding successful editing events. We also demonstrated that PVX-mediated GE can be applied to other Solanaceous crops, such as potato (*Solanum tuberosum*) and eggplant (*Solanum melongena*). These simple and highly efficient VIGE methods have great potential for generating genome-edited plants in Solanaceous crops.

## Introduction

The development of new genetically modified (GM) crops can be strongly affected by regulatory approval from governmental authorities. Indeed, these approval systems are designed to prevent harm to human health and the environment, while fostering consumer confidence in the biosafety of GM crops. However, the regulatory requirements for obtaining GM approval can be extremely time-consuming and their associated costs high, resulting in delays in product deployment and marketing. Genome editing (GE) technology may offer an alternative, as demonstrated by the approval of a genome-edited mushroom in the United States in 2016. This edited mushroom was exempt from regulation because it did not contain foreign DNA typically introduced into the genome of GM crops by traditional transgenesis, thus making it fall outside the legislation of GM organisms [1–6]. Additionally, the Food and Drug Administration has approved a drought-tolerant soybean (*Glycine max*) and false flax (*Camelina sativa*) with increased oil content, demonstrating that genome-edited crops may not require the same stringent regulations as traditional GM crops.

The use of the clustered regularly interspaced short palindromic repeats (CRISPR)/CRISPR-associated nuclease (Cas) system has the potential to revolutionize crop breeding [2, 7, 8]. The CRISPR/Cas system has been widely deployed for mutagenesis in various plant species. Plant GE using CRISPR/Cas is primarily achieved via *Agrobacterium* (*Agrobacterium tumefaciens*)-mediated introduction of a transgene encoding Cas and expressing a single guide RNA (sgRNA) (Fig. S1A). Although transgenes can be segregated out during later generations, this is labor-intensive and time-consuming [9]. Furthermore, genome-edited plants developed through *Agrobacterium*-mediated transformation continue to express *Cas* and the sgRNA even after initial GE, which may increase off-target effects [10]. Accordingly, various transgene-free GE methods are being developed with transient expression of CRISPR/Cas reagents to bypass or prevent these issues. For example, transfection of a Cas-sgRNA ribonucleoprotein complex into protoplasts or fertilized zygotes has been primarily deployed in animals and model crops [11, 12] (Fig. S1B and C). Importantly, the applications of these methods to crop plants are limited, as it is difficult to isolate protoplasts and regenerate entire plants from them. Biolistic bombardment and electroporation (Fig. S1B and C) directly introduce Cas and sgRNA into zygotes or embryos to introduce targeted mutations, although they typically do so with low editing efficiency [13, 14]. Diverse studies have been conducted to obtain transgene-free genome-edited plants, and the efficacy of genome-edited events has recently increased via RNP treatment in protoplasts. However, tissue culture requires a lengthy regeneration period from protoplasts to entire plants [15]. Since most genome-edited plants go through some tissue culture steps, only species and genotypes with robust regeneration protocols can be produced via this method. Efforts have been undertaken to circumvent the requirement of tissue culture by specifically targeting meristem or egg cells for CRISPR/Cas reagents delivery. Recent studies have prioritized the enhancement of transformation efficiency through the utilization of morphogenic genes, the establishment of phytohormone-free tissue culture protocols, and the implementation of direct plant transformation techniques, including in planta transformation [16–21]. These advanced approaches aim to optimize the effectiveness and efficiency of GE while minimizing dependence on conventional tissue culture methods.

Virus-induced genome editing (VIGE) was developed to overcome these limitations. The VIGE system is divided into two approaches: 1) delivering sgRNA to transgenic plants expressing *Cas* through a plant virus, and 2) simultaneously delivering *Cas* and the sgRNA through a plant virus with a large cargo capacity. The delivery of a sgRNA into *Cas9* transgenic plants via VIGE was first attempted in *Nicotiana benthamiana* using a viral vector based on *tobacco rattle virus* (TRV) [22]; however, the heritability of the resulting somatic mutations was extremely low (1 mutant/438 seedlings). Several studies have reported increased somatic and heritable mutations in *N. benthamiana* and Arabidopsis [22–26] using viral systems based on TRV, *cotton leaf crumple virus* (CLCrV), and *barley stripe mosaic virus* (BSMV) expressing sgRNAs attached to mobile RNA sequences, such as sequences derived from Arabidopsis *FLOWERING LOCUS T* (*FT*) and tRNA-Ile. Moreover, the TRV-mediated gene editing system has shown successful heritable gene knockouts and base editing in *Arabidopsis* [27]. Notably, tissue culture-free edited plants were also achieved using BSMV and CLCrV in *Cas9*-overexpressing barley and cotton, respectively. These advances indicate the continuous expansion of the application possibilities for this versatile VIGE system [28, 29]. However, the utilization of the VIGE system has primarily been limited to model plants like *N. benthamiana* and *Arabidopsis* [22, 30]. Although there have been successful applications in food and industrial crops such as barley and cotton, its extension to horticultural crops like tomatoes, eggplants, and potatoes has not been reported in the literature [28, 29]. Since TRV and PVX have a broad host range, deployment of the TRV- and PVX- mediated GE system to other crops, such as tomato (*Solanum lycopersicum*), will enable highly efficient, simple, and speedy GE. Unlike the TRV-mediated GE system, which cannot carry large inserts such as that of *Cas9*, *potato virus X* (PVX), *sonchus yellow net rhabdovirus*, and *tomato spotted wilt virus* (TSWV) can handle large cargo size, which allows for simultaneous expression of *Cas9* and sgRNA from a single virus-based vector [31–33]. These plant RNA viruses have a flexible filamentous structure with an RNA genome and can autonomously replicate in plants to deliver *Cas9* and the sgRNA [34–36].

In this study, we present two VIGE methods for Solanaceous crops using TRV and PVX viral systems. As a proof of concept, we targeted *Phytoene desaturase* (*PDS*) and obtained efficient edited events in *PDS*. We increased the editing efficiency of these systems by subjecting explants to a heat treatment in both systems. In our VIGE approach, antibiotic selection was not conducted during tissue culture, thereby reducing the tissue culture duration by approximately two months compared to conventional Agrobacterium-mediated transformation methods (Fig. S2). Furthermore, we successfully edited two other Solanaceous crops, potato (*Solanum tuberosum*) and eggplant (*Solanum melongena*), with the PVX-mediated VIGE system. These efficient VIGE methods will be valuable for functional genetic studies and breeding programs in Solanaceous crops.

## Results

### Optimization of the TRV-mediated GE system in tomato

We generated transgenic tomato plants expressing *Cas9* by transforming the tomato cv. Micro-Tom (MT) with a modified pHSE401 vector, in which the *cauliflower mosaic virus* (CaMV) *35S* promoter was replaced by the maize *Ubiquitin* (*Ubi*) promoter (Fig. S3A). We confirmed *Cas9* expression by reverse-transcription PCR (RT-PCR) in the first generation (T_1_) after selecting transgenic T_0_ plants by PCR (Fig. S3B, C and Table S1). We also used vector-specific primers to check for contamination of plants by *Agrobacterium* harboring the vector in the T_0_ plants (Table S1 and Fig. S3B). We obtained *Cas9* T_3_ homozygous lines, of which we chose Line 6 for TRV-mediated GE (Fig. S3C).

To optimize promoter expressing sgRNA in TRV GE system, we conducted experiments using both the plant-specific *U6–26* and Pea early-browning virus (PEBV) promoters for sgRNA expression in *Nicotiana benthamiana,* because *4′OMT2* was efficiently knocked-out in opium poppy (*Papaver somniferum* L.) using TRV-mediated GE system [37]. We observed comparable editing efficiency, and in fact, better results were achieved with the *U6-26* promoter in the systemic editing and mutant ratio of regenerants (Fig. S4). Consequently, we opted to use the *U6-26* promoter over the viral-specific PEBV promoter in subsequent studies.

**Figure 1:**
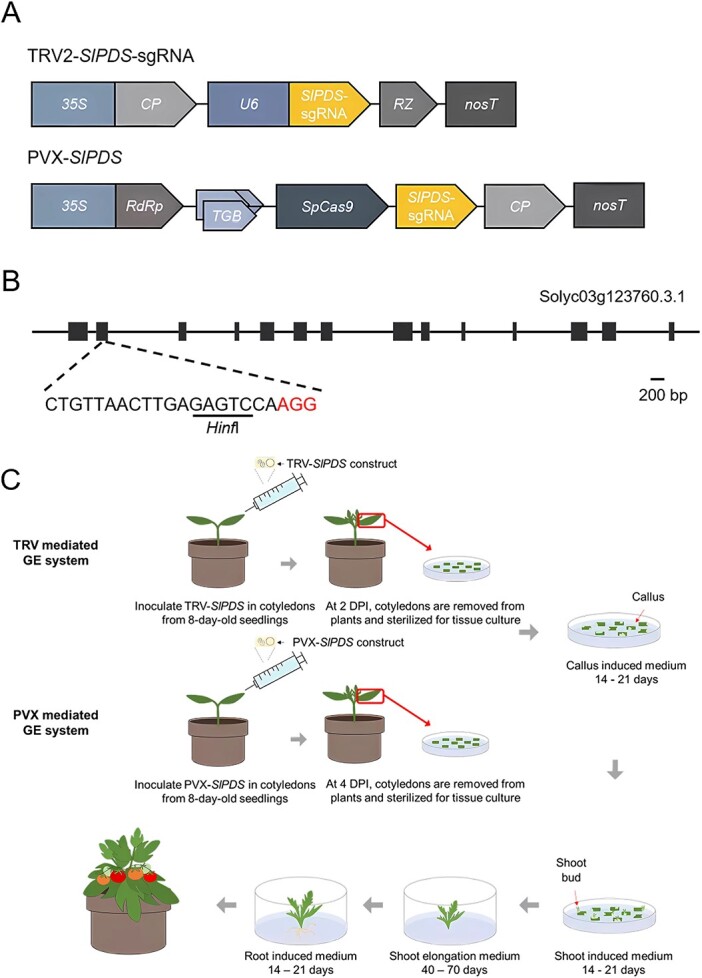
Diagram of the VIGE system in tomato. **A** Diagrams of the TRV2-sgRNA and PVX VIGE constructs targeting *SlPDS.* In the TRV2 vector, the *SlPDS*-sgRNA was cloned downstream of the *U6–26* promoter. In the PVX vector, the *SlPDS*-sgRNA was cloned downstream of *SpCas9*. *35S*, *cauliflower mosaic virus* (CaMV) *35S* promoter; *CP*, *coat protein*; *U6*, *Arabidopsis thaliana U6–26* promoter; *RZ*, *terminating ribozyme*; *nosT*, *nopaline synthase terminator*; *RdRp*, *RNA-dependent RNA polymerase*; *TGB*, *triple gene block*; *SpCas9*, *Streptococcus pyogenes Cas9*. **B** Diagram of the *SlPDS* locus and sgRNA target site. The restriction site for the enzyme *Hinf*I, used for mutation detection, is underlined in the sgRNA target sequence. **C** Overview of the TRV- and PVX-mediated GE approaches. No antibiotics were added to the growth media.

**Figure 2:**
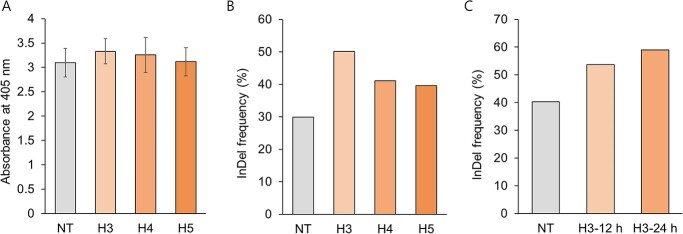
Effects of heat treatment on TRV-mediated GE of *SlPDS* in tomato. **A** Accumulation of TRV-CP in cotyledons inoculated with TRV-*SlPDS*-sgRNA and subjected to HT for 24 hours and collected at 7 DPI, as determined by ELISA. Values are means ± standard deviation (SD). n = 4–8. **B** InDel frequency after a 24-hour HT in MT inoculated with TRV-*SlPDS*-sgRNA. The InDel frequency was assessed by deep sequencing. At least five biological replicates were used for each treatment. **C** InDel frequency after a 12-hour or 24-hour HT at 3 DPI. At least eight biological replicates were used for each treatment. NT, Normal condition (23°C); H3, 37°C for 24 hours starting at 3 DPI; H4, 37°C for 24 hours starting at 4 DPI; H5, 37°C for 24 hours starting at 5 DPI; H3, 12 hours, 37°C for 12 hours starting at 3 DPI; H3, 24 hours, 37°C for 24 hours starting at 3 DPI.

To perform TRV-mediated GE in tomato, we inoculated 8-day-old *Cas9* Micro-Tom (CMT) T_3_ seedlings with the TRV-*SlPDS*-sgRNA viral vector (Fig. 1A). At 7 days post-inoculation (DPI), we extracted genomic DNA (gDNA) from TRV-inoculated cotyledons to detect mutations at *S. lycopersicum PDS* (*SlPDS*; LOC544073) through deep sequencing, resulting in an editing efficiency of 29.9%. To improve the editing efficiency, we subjected inoculated seedlings to a 37°C heat treatment (HT), corresponding to the optimal temperature for Cas9 activity. Due to the potential effects of HT on virus accumulation, we optimized the timing of HT after TRV inoculation. To this end, we inoculated CMT seedlings with TRV-*SlPDS*-sgRNA and subjected them to a HT at 37°C for 24 hours between 2 and 5 DPI. We performed an enzyme-linked immunosorbent assay (ELISA) to quantify the abundance of TRV coat protein (CP). While none of the seedlings exposed to HT at 2 DPI survived, we detected no significant difference (*p* value 0.407–0.900 in the t-test) in relative CP abundance between seedlings maintained in normal conditions and those subjected to HT from 3 to 5 DPI (Fig. 2A**),** indicating that HT does not affect the virus accumulation.

We inoculated CMT seedlings with TRV-*SlPDS*-sgRNA and subjected the explants to HT at 37°C for 24 hours between 3 and 5 DPI and investigated the effects of HT on editing efficiency by deep sequencing. Accordingly, we amplified the *SlPDS* target site from five to six heat-treated explants for each condition and pooled them for deep sequencing. Compared to the control (NT) condition (29.9% insertion/deletion (InDel) frequency), we observed the highest increase in InDels at 3 DPI (50.2%), followed by 4 DPI (41.2%) and 5 DPI (39.6%) (Fig. 2B). To present statistics, the Sanger sequencing data, ab1 file of the same samples were used for Deconvolution of Complex DNA Repair (DECORD) v3.0 tool. InDel frequency of H3 significantly increased compared to NT (Fig. S5A). HT at 3 DPI therefore resulted in the highest editing efficiency, although some explants suffered from tissue necrosis during tissue culture after HT.

To minimize tissue necrosis following HT, we tested the effects of a shorter HT of 12 hours. Notably, editing efficiency increased by 33% and 46% (from 40.3% to 53.7% and 59.0%, respectively) at 3 DPI after a 12-hour or 24-hour HT, respectively, compared to normal conditions (Fig. 2C). The result using DECORD tool also showed that editing efficiency significantly increased as HT time increasd (Fig. S5B). At 3 DPI, 24 hours of HT increased editing efficiency, but it led to necrosis in all explants. However, when the HT was reduced to 12 hours, necrosis was lower compared with 24 hours treated explants, and the redifferentiation efficiency was not significantly different from that of NT explants. Consequently, we successfully established the optimal conditions, and the 12-hour heat treatment on the media resulted in similar redifferentiation rates **(Data not show)** with editing efficiency increasing by 33% compared to NT explants (Fig. 2C). When necrosis does not significantly affect redifferentiation, the HT method that can increase the GE is effective strategy for obtaining edited plants. Therefore, we established the optimal condition of a 12-hour HT at 3 DPI. To compare the GE efficiency with NT condition, tissue culture was performed under two conditions: NT and 12 hours HT at 3 DPI. These results indicate that shortening the HT to 12 hours enhanced editing efficiency while minimizing tissue damage. Furthermore, we detected no difference in the pattern of insertions, deletions, or substitutions at the *SlPDS* target site under normal conditions and HT (Fig. S6A–D), aside from an overall increase in the mutation rate.

### Testing mobile sgRNAs in the TRV-mediated GE system

To achieve heritable gene editing in tomato, we designed TRV-mediated GE vectors by incorporating mobile sequences from *FT* or tRNA-Ile in the sgRNAs (Fig. S7). As the *SlPDS* target site destroys a restriction site for *Hinf*I, we turned to a cleaved-amplified polymorphic sequence (CAPS) analysis using gDNA extracted from systemic leaves at 70 DPI to test editing in these leaves. However, we could not detect clearly undigestable amplicons, indicating that mobile sgRNAs were ineffective in inducing genome editing in systemic leaves in tomato (Fig. S8).

### Development of *SlPDS*-edited lines through the TRV-mediated GE system

**Figure 3:**
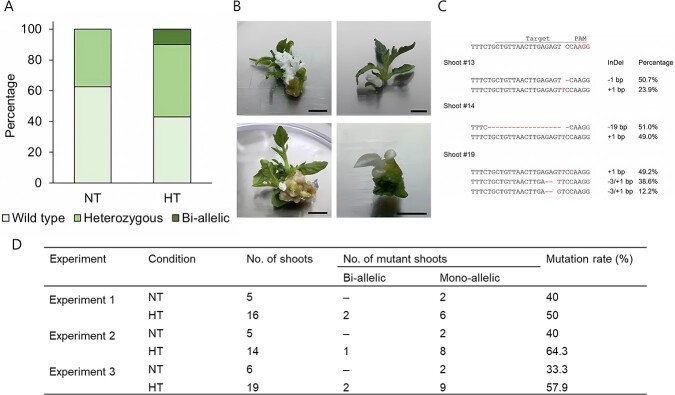
Regeneration of *SlPDS*-edited plants through TRV-mediated VIGE in tomato. **A** Percentage of regenerated shoots carrying edited sequences from MT plants inoculated with TRV-*SlPDS* at normal temperature (23°C; NT, n = 16) or exposed to a 12-hour HT (HT, n = 49). **B** Representative phenotypes of regenerated bleached shoots carrying bi-allelic mutations at *SlPDS*. **C** Sequences of the *SlPDS* target region in regenerated bleached E_0_ mutants and percentages of each mutation type. **D** Summary of the mutation rate in regenerated shoots from NT and HT explants.

To develop edited tomato plants at *PDS* using the TRV-mediated GE system, we inoculated CMT cotyledons with the TRV-*SlPDS*-sgRNA viral vector, followed by tissue culture. At 3 DPI, we treated the explants with HT at 37°C for 12 hours before initiating regeneration by tissue culture. Without HT, we obtained a mutation rate of 37.5% in regenerated shoots, compared to 57.1% for shoots regenerated from heat-treated explants (Fig. 3A), demonstrating a higher mutation rate in the shoots regenerated from heat-treated explants.

In addition, HT resulted in a high proportion of bi-allelic mutants, accounting for 10.2% of all genotyped regenerated shoots, whereas bi-allelic mutations were absent from shoots regenerated from explants not exposed to HT. Notably, some of the bi-allelic mutants led to bleached shoots as a results of frame-shift mutations, as shown in Fig. 3B. Analyzing the *SlPDS* sequence around the sgRNA target site in shoots regenerated from heat-treated explants revealed various InDels, providing further evidence of successful gene editing (Fig. 3C). To detect mutations in the regenerated shoots, we conducted a CAPS analysis with the same marker as above. We detected a mutation rate of 33.3–40% in the control explants not exposed to HT (NT), which rose to 50–64.3% in explants subjected to HT (Fig. 3D). We sequenced gDNA extracted from 28 regenerated shoots and identified 1-bp insertions in 17 shoots (60.7%), bi-allelic mutations in four shoots (14.3%), and multiple mutations in two shoots (7.1%) (Fig. S9).

**Figure 4:**
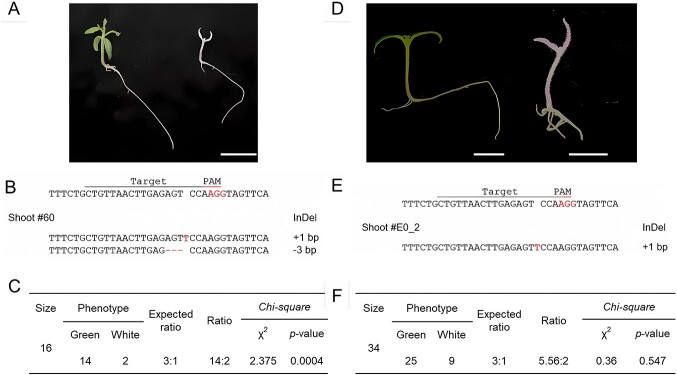
Inheritance analysis of VIGE-mediated editing of *SlPDS* in the E_1_ generation in tomato. **A–C** Representative phenotypes of E_1_ plants generated by TRV-mediated GE and number of plants showing a wild-type (green) or mutant (white) phenotype and genotype. **A** Phenotype of *SlPDS* E_1_ mutant plants regenerated from shoot #60. Scale bar, 2 cm. **B** Sequence of the *SlPDS* target region in shoot #60 mutant. **C** Segregation of the albino phenotype in E_1_ mutant plants derived from shoot #60. **D–F** Representative phenotypes of E_1_ plants generated by PVX-mediated GE and number of plants showing a wild-type (green) or mutant (white) phenotype and genotype. **D** Phenotype of E_1_ mutant plants derived from the E_0_–2 edited event carrying mutations at *SlPDS*. Scale bar, 1 cm. **E** Sequence of the *SlPDS* target region in shoot # E_0_–2 mutant. **F** Segregation of the albino phenotype in E_1_ mutant plants derived from the E_0_–2 edited event.

We investigated the stable inheritance of gene edits by characterizing the phenotype and genotype of the Edited 1 (E_1_) generation (Fig. 4A-C). To this end, we sowed E_1_ seeds derived from shoot #60 on half-strength Murashige and Skoog (MS) medium and sequenced the *SlPDS* target site. Seedlings displaying a photobleached phenotype were homozygous for a 1-bp insertion (Fig. 4A). Out of 16 seedlings, we identified two photobleached seedlings, and 14 green seedlings (Fig. 4B). Notably, the seedlings carrying one or two copies of *SlPDS* with a 3-bp deletion were green (Fig. 4C), indicating that the 3-bp deletion does not affect SlPDS function, likely because it maintains the reading frame. In our investigation, we conducted RT-PCR tests to confirm whether TRV is transmitted to the next generation. The result demonstrated the absence of TRV coat protein expression in the E_1_ seedlings (Fig. S10). These results suggest that the TRV-mediated GE system induces heritable editing without TRV seed transmission.

**Figure 5:**
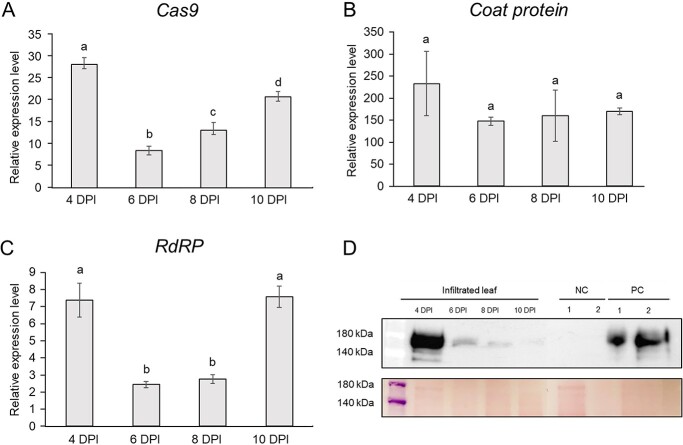
Confirmation of foreign gene expression in *S. lycopersicum* cv. MT inoculated with the PVX-*SlPDS* vector. **A–C** RT-qPCR analysis of transient expression of *Cas9* (**A**), the coat protein gene (**B**), and *RdRP* (**C**). Cotyledons were collected from seedlings inoculated with PVX-*SlPDS* every 2 days from 4 days post inoculation (DPI) to 10 DPI. *SlActin* was used as the internal reference. Three biological replicates were used for each condition. **D** Immunoblot analysis of MT cotyledons inoculated with the PVX-*SlPDS* construct. Cas9 was detected using Cas9-specific antibodies. Samples were collected from MT cotyledons inoculated with PVX-*SlPDS* every 2 days from 4 to 10 DPI. NC, negative control (MT infiltrated with infiltration buffer); PC, positive control (Cas9-positive transgenic MT). Values are means ± SD. Different lowercase letters indicate a significant difference according to Tukey’s test (*P* < 0.05).

### Optimization of the PVX-mediated GE system in tomato

As TRV cannot handle large inserts like *Cas9*, we generated another virus vector based on PVX that included the sgRNA and *Cas9* in the same construct. In this study, the PVX vector, generously provided by Kazuhiro Ishibashi (Institute of Agricultural and Biological Sciences, Tsukuba, Japan), was utilized. The vector constructs and sequences remained consistent with those detailed in Ariga et al. (2020) [31], with the exception of the sgRNA sequences, which were modified for our study. We then assessed the expression of genes delivered by the PVX vector: *Cas9*, *PVX-CP*, and *RNA-dependent RNA polymerase* (*RdRp*), using total RNA extracted from MT cotyledons inoculated with PVX-*SlPDS* targeting *SlPDS*. Tissue culture is a labor-intensive process, making it crucial to identify the most efficient conditions for gene editing. To achieve this, we conducted several experiments to determine the optimal conditions. Real-Time quantitative PCR (qRT-PCR) analysis detected the expression of *Cas9*, *PVX-CP*, and *RdRP* in these inoculated MT cotyledons (Fig. 5A–C). These genes were most highly expressed at 4 DPI, before decreasing at 6 DPI and rising again until 10 DPI, possibly due to the effects of surface sterilization of inoculated leaves before tissue culture on the virus accumulation. If the cotyledon is separated from the plant, cell division may not be as active, which can affect the virus accumulation. However, immunoblot results indicated a gradual decline in Cas9 protein levels, particularly noticeable at 10 DPI, where the protein became barely detectable (Fig. 5D). The Cas9 protein functions by binding to the gRNA, cleaving the target site, and subsequently undergoing degradation [38]. Consequently, the explant at 10 DPI, exposed to the optimal active temperature of the Cas9 protein due to a heat treatment performed at 9 DPI, would have been heavily used for gene editing at the optimal active state of the Cas9 protein. Subsequently, the protein would have degraded, leaving only a minimal amount detectable. We also detected *Cas9* expression in systemic MT leaves at 7 DPI (Fig. S11). We confirmed Cas9 accumulation in MT cotyledons and systemic leaf inoculated with PVX-*SlPDS* by immunoblot analysis with an anti-Cas9 antibody (Fig. 5D and Fig. S11B).

**Figure 6:**
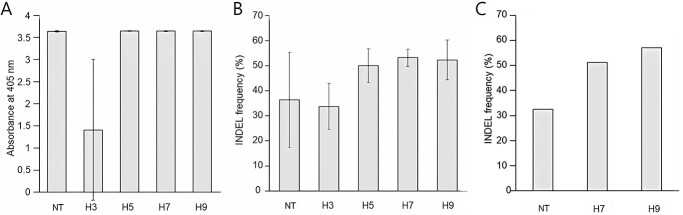
Effects of HT on PVX-mediated GE of *SlPDS* in tomato. **A** PVX-CP accumulation in cotyledons inoculated with PVX-*SlPDS*, as determined by ELISA at 10 DPI. **B** InDel frequency after a 24-hour HT in MT inoculated with PVX-*SlPDS*. A CAPS analysis was performed to estimate InDel frequency. DNA band intensities in the gel image were quantified by ImageJ software. **C** InDel frequency, calculated based on deep sequencing results from seedlings inoculated with sgRNA-*SlPDS* constructs. Three biological replicates were used for each treatment. C, Normal condition (23°C); H3, 37°C for 24 hours starting at 3 DPI; H5, 37°C for 24 hours starting at 5 DPI; H7, 37°C for 24 hours starting at 7 DPI; H9, 37°C for 24 hours starting at 9 DPI. Values are means ± SD. Three biological replicates were used for each condition.

We investigated the effects of HT on PVX accumulation by conducting an ELISA to quantify PVX-CP abundance. We observed a drop in PVX-CP in cotyledons exposed to HT at 3 DPI (H3) relative to NT seedlings, but PVX-CP levels increased following HT at 5 DPI (H5) onward (Fig. 6A). These findings suggest that HT applied at or after 5 DPI may help support the spread of PVX.

To investigate the effects of HT on editing efficiency, we treated MT cotyledons with HT every 2 days from 3 to 9 DPI and collected all treated explants at 10 DPI for gDNA extraction and CAPS analysis. We observed no clear differences between NT and H3 (HT applied at 3 DPI) explants (Fig. S12A). CAPS analysis was conducted using the same amount of PCR product and digested with the restriction enzyme that recognize the target site. In explants exposed to HT at and after 5 DPI (H5, H7, H9), the editing efficiency increased compared to the control, as determined by InDel frequency (Fig. 6B). Indeed, editing efficiency increased by 47.5% in the H7 explants and by 43.5% in the H9 explants relative to NT explants (Fig. 6B), indicating that the editing efficiency improved when imposing HT at 7 and 9 DPI. When the HT exposed explants and the normal condition explants were transferred for tissue culture 10 DPI, only calli was formed, and no shoots were induced **(Data not shown)**. Although younger cotyledons showed improved differentiation, it was necessary to optimize the regeneration conditions while considering the potential effects on virus spread and editing efficiency. Based on the higher abundance of PVX-CP at 5 DPI and thereafter (Fig. 6A), we chose 4 DPI as the optimal time to surface-sterilize the explants and initiate tissue culture.

In a second experiment, we aimed to increase the regeneration efficiency while maintaining high editing efficiency. For this purpose, we surface sterilized the inoculated cotyledons at 4 DPI and subjected them to HT at 7 or 9 DPI, after which we used the CAPS marker and deep sequencing to evaluate editing efficiency. The CAPS analysis revealed an undigested band in control explants not exposed to HT (NT), as well as in explants exposed to HT at 7 (H7) and 9 DPI (H9), indicative of editing (Fig. S12B). Deep sequencing analysis indicated that editing efficiency was higher in heat-treated plants by 57.6% (H7) and 75% (H9) compared to NT explants (Fig. 6C). We also examined the mutations at the *SlPDS* target site by analyzing insertions, deletions, and substitutions in the deep sequencing data: while HT can increase editing efficiency, it did not alter the pattern of sequence variation (Fig. S13A–D). Therefore, we selected 24 hours HT at 7 DPI and 9 DPI as the optimal conditions. To compare the GE efficiency with NT condition, tissue culture was performed under three condition: NT, 24 hours at 7 DPI and 9 DPI, respectively.

### Obtaining transgene-free targeted mutagenesis in MT via PVX-mediated GE

**Figure 7:**
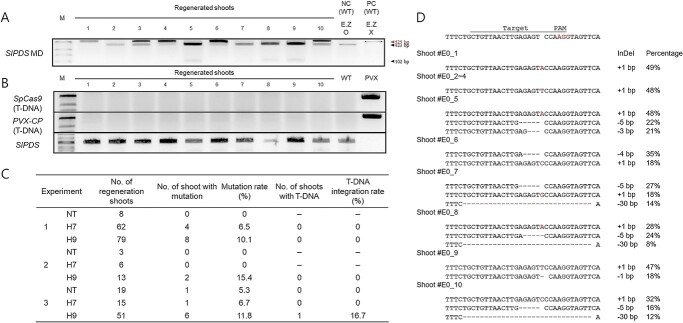
Transgene-free genome editing in tomato using PVX-mediated GE through tissue culture. **A** Mutation detection (MD) through CAPS analysis of regenerated *SlPDS*-edited shoots. A CAPS analysis was conducted to identify edited shoots from the explants inoculated with PVX-*SlPDS*. The *SlPDS* sequence with mutations in the *Hinf*I target site are not cleaved by the *Hinf*I enzyme (525 bp), whereas the wild-type *SlPDS* sequence is cleaved into two fragments (423 bp and 102 bp). E.Z O, incubated with *Hinf*I; E.Z X, not incubated with *Hinf*I. **B** Transgene-free genome editing using a PVX vector. PCR was performed to detect the presence of the transgene derived from the PVX-*SlPDS* construct. *Cas9* and *CP* primers were designed based on the PVX-*SlPDS* plasmid sequence, and the PVX-*SlPDS* plasmid was used as a positive control, and MT (wild type) gDNA was used as a negative control. *SlPDS*-specific primers were designed from the *SlPDS* reference sequence, with MT (wild type) gDNA used as a positive control, and the PVX-*SlPDS* plasmid used as a negative control. **C** Summary of mutations detected in regenerated shoots based on the CAPS analysis. Mutation rates were calculated from three independent experiments. **D** Sequence of the *SlPDS* target region in regenerated mutant plants and percentage of each mutation type.

To develop edited tomato plants at *SlPDS* using the PVX-mediated GE system, we inoculated cotyledons with the PVX-*SlPDS* vector and transferred the explants to tissue culture. Previous study has shown that the efficiency of genetic editing is enhanced when *Agrobacterium* transformed with PVX vectors is introduced into *N. benthamiana* leaves, as compared to the use of vectors that transiently express *SpCas9* and *sgRNA*. This result implies that, even when *Agrobacterium* is employed for the delivery of PVX vectors, the editing process is mediated by viruses rather than T-DNA integration [31]. Consequently, this method qualifies as a transgene-free genetic editing approach. We identified edited plants by CAPS analysis and sequencing. Using the CAPS marker, the PCR products from wild-type regenerated shoots were completely cleaved upon digestion with the corresponding restriction enzyme. However, we detected undigested PCR products in shoots regenerated from cotyledons inoculated with PVX-*SlPDS*, indicating successful GE (Fig. 7A). The *SlPDS* sequence with mutations in the *Hinf*I target site is not cleaved by the *Hinf*I enzyme (525 bp), whereas the wild-type *SlPDS* sequence is cleaved into two fragments (423 bp and 102 bp). Notably, not all edited shoots showed a fully undigested PCR amplicon, suggesting bi-allelic or mosaic mutations. We analyzed the editing efficiency in 256 regenerated shoots with the CAPS marker, resulting in an editing efficiency of 1.77% without HT, and 4.4% and 12.4% with HT applied at 7 and 9 DPI, respectively (Fig. 7C). These findings indicate that HT increased the editing efficiency by 149% and 708% at H7 and H9, respectively, compared the control. Consistent with these results, Sanger sequencing revealed that edited regenerated shoots carry mutations at the target region, in contrast to the wild-type regenerated shoots (Fig. 7D). Sequencing of eight shoots identified two shoots (25%) with a 1-bp insertion, while the remaining six shoots (75%) harbored multiple mutations (Fig. 7D).

To check for T-DNA integration, we used *Cas9-* and *PVX-CP*-specific PCR primers to genotype E_0_ plants with confirmed *SlPDS* editing by CAPS analysis. Among 22 E_0_ plants genotyped, one plant (4.5%) appeared to carry the vector in its genome (Fig. 7B and C), indicating that the PVX VIGE system can sometimes result in transgene integration. To exclude such plants, all edited plants should be genotyped to confirm that they are free of transgenes.

### Obtaining bi-allelic mutants from E_1_ plants through PXV-mediated GE

To generate E_1_ lines, we selected the E_0_–2 plant among the *SlPDS* mutants and allowed it to self-pollinate. We sowed the resulting seeds on half-strength solid MS medium; notably, some E_1_ seedlings exhibited bleaching phenotypes (Fig. 4D). We extracted gDNA from the cotyledons of E_1_ plants to conduct a CAPS analysis. Among 34 seedlings, nine were bleached and 25 were fully green (Fig. 4E). The CAPS marker revealed that all nine bleached seedlings lack the wild-type sequence at *SlPDS*, while 18 green seedlings were heterozygous. The remaining seven green seedlings showed the fully digested PCR amplicon, indicating that they carry an intact *SlPDS* copy (Fig. 4F). These results were consistent with a Mendelian segregation ratio for a recessive single mutation, suggesting that the mutation is stable and heritable, with the potential to be transmitted to subsequent generations.

### PVX-mediated targeted mutagenesis in other Solanaceous crops

To explore the applicability of PVX infection in other Solanaceous crops, we inoculated 11 potato cultivars and 10 eggplant cultivars with PVX-Green fluorescence (GFP) (Fig. S14). After ELISA analysis, we selected cultivars susceptible to PVX infection based on PVX-CP accumulation: ‘Desiree’ for potato and ‘Jeonbuk Gochang’, ‘Baekgagi’, and ‘Chungbuk Youngdong’ for eggplant. We then constructed PVX VIGE vectors targeting *PDS* in potato (*StPDS*; LOC102577582) or eggplant (*SmPDS*; LOC101244544) and inoculated the resulting VIGE constructs into the true leaves of potato plants and eggplant cotyledons via *Agrobacterium* as described above for tomato.

Following inoculation, we subjected potato and eggplant explants to HT at 9 DPI, which was the most effective in tomato, and collected all explants for CAPS analysis. We detected editing events in all groups, with editing efficiency for explants subjected to HT increasing by 22.1% to 30.5% in potato and by 22.0% to 116.1% in eggplant compared to the controls (Fig. S15). For the tetraploid potato, we confirmed the edited sequences and editing efficiency through sequencing. Consistent with the CAPS analysis, we observed that HT increased editing efficiency by 22.2 to 50 times, and we could see a wide range of variants occurring (Fig. S16). We detected varying editing efficiencies for potato and eggplant depending on the specific sgRNA used (Fig. S15 and Fig. S16). Additionally, we developed a gRNA by identifying a conserved region in the *PDS* gene of both potato and eggplant (*St&mPDS1*). We observed successful editing in the inoculated leaves using a PVX vector containing the same gRNA. These results serves as evidence that gene editing can be achieved in various Solanaceous plants through the PVX vector system in addition to tomato.

## Discussion

Tomato is widely grown and consumed worldwide due to its economic benefits and high nutritional value. Numerous studies have reported the use of GE based on the conventional transgene-mediated methods to explore the mechanisms behind tomato domestication, fruit quality, resistance to biotic stress, and tolerance to abiotic stresses [39–42]. VIGE has been developed to overcome imitations of these traditional methods. However, successful example of VIGE in tomato has not yet been reported, except for VIGE with TSWV^33^. In the case of the TSWV-mediated VIGE system, they employed ribavirin chemotherapy to prevent the virus from spreading to the regenerating cells, owing to the virus’s robust virulence, broad host range, and the potential environmental risks associated with its release [33]. Additionally, the use of leaves with strong pathogenicity could affect the tissue culture process [33]. In contrast, the VIGE system we have developed, utilizing TRV and PVX, exhibits a milder pathogenicity compared to TSWV and is relatively less infectious [22, 30, 31, 33]. Both viruses exhibit reduced effects compared to TSWV, both in terms of pathogenicity and their impact on plant growth. As a result, they are frequently employed in virus-induced gene silencing systems [43–45]. Consequently, it can be conveniently employed in tissue culture without the need for the aforementioned treatment. While the TSWV study indicated an absence of phytotoxicity in tobacco associated with ribavirin use, it has been reported to elicit phytotoxic responses in various other crop species [33, 46, 47]. Another notable advantage lies in the streamlined vector requirements of PVX-mediated GE, which necessitates only one vector, and TRV-mediated GE, which requires just two vectors [30, 31, 43]. In contrast, TSWV-mediated GE demands the deployment of three vectors, underscoring the efficiency gains associated with the former systems [33]. Our study represents a notable advancement in genome editing of Solanaceous crops including tomato, potato, and eggplant. By developing two VIGE methods tailored for Solanaceous plants, we have addressed the limitations of conventional transgene-mediated approaches, which often involve time-consuming tissue culture steps to generate stable transgenic lines expressing the Cas9/sgRNA reagent. In our first approach, we successfully utilized the TRV vector to deliver sgRNAs into a transgenic tomato line (*S. lycopersicum* cultivar Micro-Tom) expressing Cas9. While previous studies utilized *Cas9* transformants driven by the 35S promoter, we developed and employed a *Cas9* transgenic tomato line with enhanced *Cas9* expression by utilizing the Maize *Ubi* promoter, known for its higher expression activity compared to the 35S promoter in both monocot and dicot plants [48, 49]. We achieved a mutation rate of 40.3%, demonstrating effective genome editing. Building on this success, we further developed a transgene-free GE method using a PVX vector to deliver both Cas9 and sgRNAs, resulting in a mutation rate of 36.5%. To optimize editing efficiency, we introduced a 37°C heat treatment, which led to notable improvements. Additionally, we successfully demonstrated the versatility of the PVX-mediated GE method by applying it to other Solanaceous crops, including potato (*Solanum tuberosum*) and eggplant (*S. melongena*). This showcases the potential broad applicability of our VIGE methods in Solanaceous crops. By overcoming the limitations of traditional methods and achieving notable editing efficiencies, we provide a valuable tool for Solanaceous crop breeding and trait improvement.

In this study, to set up protocols for VIGE in the Solanaceae crops, we attempted to detect mutations in the target gene in plants that were inoculated to deliver gRNA, and then we attempted tissue culture to obtain edited plants. However, since tissue culture is a time-consuming and laborious process, it is necessary to maximize editing efficiency before tissue culture. The CRISPR/Cas9 system is derived from the immune system of the bacterium *Streptococcus pyogenes*, and the optimal temperature for the nuclease activity of Cas9 is 37°C. Indeed, HT improves the editing efficiency of Cas9 or other Cas enzymes in plants and animals [34, 35, 50]. Recently, our group reported increased editing efficiency of PVX-mediated GE by applying a HT of 37°C in *N. benthamiana* [36], suggesting that HT can be incorporated into the VIGE system for Solanaceous crops. To enhance editing efficiency, we exposed virus-inoculated tomato cotyledons to a HT at 37°C. Even though VIGE was recently reported to be enhanced by low temperature, there was no direct comparison between low temperature of 18°C and normal conditions of 24–25°C or between low temperature and heat treatment [27]. In the case of VIGS, enhanced TRV replication and persistence due to low temperature treatment may increase VIGS efficiency, but in the case of VIGE with TRV, increased sgRNA expression through virus accumulation is possible, but the Cas9 activity in *Cas9-*transgenic plants under normal temperature conditions can greatly affect editing efficiency, so increasing the efficiency of the Cas9 protein itself through high temperature may also be a way to increase editing efficiency. In addition, since this experiment was not a case of systemic or heritable editing, but rather the acquisition of mutants through tissue culture in inoculated leaves, we did not focus on virus systemic spread and persistence, but rather on the effect of increasing editing efficiency through high temperature treatment in inoculated leaves. The heat treatment did not affect virus accumulation, but only increased editing efficiency. The HT did not affect virus accumulation in the TRV system, but appeared to transiently limit virus accumulation in the PVX system at 3 DPI (Fig. 2A and Fig. 6A). Shoots regenerated from inoculated explants revealed that editing efficiency increased in TRV- and PVX-mediated GE by 33–46% and 56–76%, respectively, after HT (Fig. 2C and Fig. 6C). These results suggest that HT enhances the editing efficiency in the tomato VIGE system. We detected the typical type of small nucleotide deletions, substitutions, and insertions induced by CRISPR/Cas9 regardless of HT exposure (Fig. S5**and 13)**. We conclude that HT does not affect the mutation pattern but increases editing efficiency, thus optimizing the conditions for highly efficient GE in both VIGE systems.

During the process of VIGE, there have been some cases where the virus infects the seeds of the subsequent generation or where transgenes become integrated into the host plant genome via *Agrobacterium* [31]. In this study, we also examined the plant material obtained from this experiment to determine if such issues are present. In TRV-mediated GE, we confirmed no TRV seed transmission to the next generation through TRV-CP RT-PCR. In *N. benthamiana* and *Arabidopsis*, previous reports also indicate no seed transmission of viruses in TRV-mediated GE systems [23, 25]. Although we did not test seed transmission of PVX, previous studies consistently reported that PVX does not transmit through seeds in tomatoes [51, 52]. In the case of *N. benthamiana*, there are also reports of no seed transmission of viruses in PVX-mediated GE systems. These results show the successful inheritance of TRV- and PVX-mediated GE without any virus transmission in the next generation. However, seed transmission can be problematic in vegetative propagation crops like potatoes, where the infection is primarily transmitted through mechanical contact [53]. We also showed that some edited plants contained transgene in PVX-mediated editing (Fig. 7B and C), demonstrating that there is possibility that transgenes can be integrated into the host plant genome when using this system.

When using PVX and TRV for GE, there can be advantages and disadvantages. Firstly, in terms of efficiency, TRV-mediated GE was significantly higher compared to GE using PVX-mediated GE. Using the TRV-based system, we obtained 34 regenerated shoots with editing at *SlPDS*. The editing efficiency increased by 52% after 12 hours of HT compared to normal conditions (Fig. 3A and D). In the PVX-mediated GE system, we obtained 22 shoots with edits at *SlPDS* out of 252 regenerated shoots. The mutation rate was 3.1% under normal conditions, and 6.1% - 10.2% after HT (Fig. 7C). This is attributed to the continuous expression of the Cas9 protein in the case of TRV-mediated GE, where editing occurs immediately once the sgRNA is expressed by TRV. In the case of PVX-mediated GE, both the Cas9 protein and sgRNA are expressed simultaneously, potentially leading to efficiency, but the large size of the Cas9 protein (4.8 kb) could result in the virus gradually eliminating the Cas9 gene over time. To address these issues, it may be necessary to use smaller nucleases such as Cas12b. There were also differences between the TRV and PVX systems in the types of mutations generated through GE. Regenerated shoots harboring bi-allelic mutations at *SlPDS* accounted for 10.2% of all shoots in TRV-mediated GE (Fig. 3B). By contrast, we could not obtain bi-allelic mutants in PVX-mediated system. This is likely due to the lower number of regenerated individuals obtained through PVX-mediated GE compared to the TRV-mediated system. In our VIGE methods, antibiotic selection was excluded during the tissue culture, reducing the tissue culture duration by two months compared to conventional *Agrobacterium*-mediated transformation methods (Fig. S2).

In previous studies on TRV-mediated genome editing in *N. benthaminana*, the efficiency of editing transferred to seeds was low [22]. To overcome these limitations, mobile RNA elements have been fused to sgRNAs for expression in *N. benthamiana* and Arabidopsis*,* resulting in improved germline delivery and increased heritable editing efficiencies [23, 24, 26, 30, 54, 55]. We tested that mobile sgRNAs can function in tomato, but we could not observe editing in systemic leaves, indicating that these modified sgRNAs are largely not mobile (Fig. S7 and S8). The lack of mobility observed despite including *FT* and *tRNA*-Ile in the vector in previous studies might be explained below. In the previous study, the mobility of mRNA molecules like florigen and antiforeign, observed in Arabidopsis, has not been extended to tomatoes: previous tomato grafting experiments did not support the long-distance movement of *single flower truss* (*SFT*) mRNA, the tomato *FT* homolog [56]. To address this matter, it is essential to investigate the potential conservation of mRNA mobility for florigen and antiforeign in other plant species before proceeding with the experiment. The *FT* and *tRNA* utilized in this study have been previously applied in Arabidopsis and tobacco, but their suitability for tomatoes may differ [56]. To overcome this, it is necessary to consider other genes, such as *NsCET1*, a member of the phosphatidylethanolamine-binding domain protein family, which has shown mobility in tomatoes [57]. More work is needed in using mobile sgRNAs that confer mobility in tomato. As demonstrated in previous studies, the dual virus system can also be employed for transgene-free purposes. It allows the design of viruses capable of producing Cas proteins along with gRNAs contained in viruses with large cargo sizes, enabling their utilization in future research endeavors [58]. Additionally, recent studies have attempted to improve transformation efficiency by employing morphogenic genes, establishing transformation through phytohormone-free tissue culture [19, 59], and performing plant transformation directly, such as *in planta* transformation, for obtaining shoots directly from plants, without tissue culture [16, 19].. The dual virus system may help facilitate efficient gene editing in a wider range of crop species and genotypes, particularly when one viral vector expresses CRISPR components and the other virus expresses morphogenic genes [19, 59, 60].

In conclusion, we developed two VIGE methods for Solanaceous plants. The first method involves delivering a sgRNA into a *Cas9* transgenic tomato line using the TRV vector, while the second method employs a PVX vector to deliver *Cas9* and the sgRNA to plant cells without transgene integration. While VIGE has been reported in the model plant *N. benthamiana*, our study marks the first successful application in Solanaceae crops. Although in this study we did not demonstrate the transmission of mutations through seeds without tissue culture, we have shown that regenerating tissues induced for gene editing by the virus can lead to much faster gene editing compared to conventional transgene-mediated approaches. Additionally, we present compelling evidence that the efficiency of genome editing in solanaceous crops can be enhanced through heat treatment during VIGE experiments. Moreover, our research demonstrates that a single PVX vector, equipped with gRNAs targeting conserved gene regions, can effectively induce gene editing across a wide range of Solanaceous species. These simple and efficient VIGE methods should prove useful for functional genetic studies and breeding in Solanaceous crops.

## Materials and methods

### Plant materials and growth conditions

Tomato (*S. lycopersicum*) cv. Micro-Tom (MT) and *N. benthaminana* were used for GE studies. Seed sterilized was performed as described by Yoon et al. (2020) [41]. The disinfected seeds were germinated on soil and the resulting seedlings were cultivated at 24 ± 2°C under a 16-hour light/8-hour dark photoperiod provided by cool fluorescent light bulbs (Bungarpho, FL40EX-D, 40 W, South Korea) in a walk-in chamber. Eleven potato (*S. tuberosum*) cultivars were provided by the Goryeong Agricultural Research Institute (Pyeongchang, South Korea). Ten eggplant (*S. melongena*) cultivars were provided by the National Agrobiodiversity Center (Jeonju, South Korea). The list of the potato and eggplant cultivars is given in Table 1. All plants were grown in a walk-in chamber at Seoul National University (Seoul, Korea).

**Table 1:** List of the potato and eggplant cultivars examined in this study

**Species**	**Cultivar**	**Source**
*Solanum tuberosum*	Kangsun	Goryeong Agricultural Research Institute
	Seohong	
	Eunsun	
	Saebong	
	Keunsun	
	Sumi	
	Gowon	
	Suji	
	Desi	
	Chuback	
	Desiree	
*Solanum melongena*	Jeonbuk Namwon	National Agrobiodiversity Center
	Jeonbuk Gochang	
	Dasangagi	
	Yangtomato	
	Baekgagi	
	Chungbuk Youngdong	
	Gyeonggi suwon	
	84	
	142	
	147	

### *Agrobacterium*-mediated transformation of tomato

Previous studies have shown that *Ubi* promoters yield higher editing efficiency than the CaMV *35S* promoter [20]. Therefore, the pHSE401 binary vector, in which the *35S* promoter was replaced with the Maize *Ubi* promoter from the pBUN501 vector was used to generate *Cas9* transgenic tomato (Fig. S2A). The modified pHSE401-*Ubipro:Cas9* vector was transformed into *Agrobacterium* strain GV3101 through electroporation. *Agrobacterium*-mediated transformation was performed as described by Yoon et al. (2020) [41] with some modifications (Table 2).

**Table 2:** Composition of the tomato tissue culture media used in this study

Medium	Compositions	Days
Pre-culture medium	MS, 30 g/L sucrose, 4 g/L phytagel, 1 mg/L NAA, 1 mg/L BAP, 500 mg/L timentin, pH 5.8	1
Shoot induction medium	MS, 30 g/L sucrose, 4 g/L phytagel, 2 mg/L *trans*-zeatin riboside, 1 mg/L IAA, 500 mg/L timentin, pH 5.8	14–21
Shoot elongation medium	MS, 30 g/L sucrose, 4 g/L phytagel, 1 mg/L *trans*-zeatin riboside, 1 mg/L IAA, 250 mg/L timentin, pH 5.8	40–70
Rooting medium	1/2 MS, 30 g/L sucrose, 4 g/L phytagel, 250 mg/L timentin, pH 5.8	14–21

NAA, 1-naphthaleneacetic acid; BAP, 6-benzylaminopurine; MS, Murashige and Skoog; IAA, indole-3-acetic acid

### sgRNA design and viral vector construction

A sgRNA targeting *SlPDS* previously shown to induce photobleaching phenotypes was cloned into the TRV2 and PVX vector (Yoon et al., 2020) [41] as proof of concept for VIGE. PVX vector was kindly provided by Kazuhiro Ishibashi (Institute of Agrobiological Sciences, NARO, Tsukuba, Japan). The sgRNA-*SlPDS* insert was then cloned into the TRV2 vector using *Mfe*I and *Xma*I restriction enzymes. In the case of mobile sgRNAs, the sgRNA-*SlPDS* insert was digested with *Mfe*I and *Stu*I, and the mobile sequence was digested with *Stu*I and *Xma*I and ligated together into the TRV2 vector.

To construct the PVX-sgRNA vectors (*SlPDS*, *St&mPDS1*, *StPDS2*, *StPDS3*, *SmPDS2*), the sgRNA sequence comprising 83 bp of scaffold RNA and the sgRNA for *SlPDS* was ligated between the *Mlu*I and *Sal*I sites, which are immediately downstream of the *SpCas9* stop codon (Fig. 1A). The amplicons were cloned using the restriction sites *Mlu*I and *Sal*I and ligated into the PVX vector. All viral constructs were transformed into *Agrobacterium* strain GV3101 through electroporation. The sequences of all primers used in the vector construction list are in Table S2.

### *Agrobacterium*-mediated infiltration of viral vectors

*Agrobacterium*-mediated infiltration was performed as described by Venkatesh et al. (2022) [36] with some modifications. *Agrobacterium* cultures harboring P19, TRV2-sgRNA, and TRV1 were collected and diluted with infiltration buffer, to reach an OD_600_ of 0.6. The cell suspension for each TRV2 vector was mixed with resuspended *Agrobacterium* cells carrying the TRV1 and P19 construct in a 1:1:1 ratio (v/v/v). *Agrobacterium* cultures carrying P19 and PVX-*GFP* or PVX-sgRNA (*SlPDS*, *St&mPDS1*, *StPDS2*, *StPDS3*, *SmPDS2*) were harvested and diluted with infiltration buffer as above to an adjusted OD_600_ of 0.3. After incubation at room temperature for 3 hours, *Agrobacterium* cultures harboring P19 and PVX-GFP or PVX-gRNA were mixed at a 1:1 ratio (v/v) and co-infiltrated into the lower side of leaves from 21-day-old *N. benthamiana* or 8-day*-*old MT cotyledons using a needleless 1-mL syringe (Fig. 1C).

### Virus infection and evaluation of TRV-CP and PVX-CP accumulation

Cotyledons of MT, CMT (transgenic MT harboring *Cas9*), eggplant, and potato seedlings were individually inoculated with TRV-*SlPDS*-sgRNA, PVX-*GFP*, or PVX-gRNA (*SlPDS*, *St&mPDS1*, *StPDS2*, *StPDS3*, *SmPDS2*) through *Agrobacterium*-mediated infiltration. At 7 DPI, one piece of leaf disc was analyzed by ELISA for the presence of TRV-CP following the manufacturer’s instructions (Tobacco Rattle Virus DAS ELISA set, Cat. No. 07152S, LOEWE, Sauerlach, Germany). At 10 DPI, leaf discs were analyzed by ELISA for the presence of PVX-CP following the manufacturer's instructions (Reagent Set for Potato virus X, Cat. No SRA10002/0500; Agdia, Elkhart, USA). ELISA was performed with at least three replications. The absorbance of the samples at 405 nm was measured using a microplate photometer (MULTISKAN FC; Thermo Fisher Scientific, USA). GFP was visualized using a confocal scanning microscope (SP8X, Leica, Germany) and a fluorescence imaging system (FOBI, Nanoscience, Korea) at 10 DPI. The confocal image analysis was performed at the National Instrumentation Center for Environmental Management (NICEM), Seoul National University, Seoul. To confirm TRV seed transmission in the E_1_ progenies, RT-PCR was conducted. The RT-PCR primer sequences are listed in Table S2.

### Extraction of nucleic acids

gDNA was extracted from inoculated cotyledons at 10 DPI and regenerated plants using a modified cetyltrimethylammonium bromide (CTAB) method [61]. Systemic leaves from seedlings inoculated with TRV-*SlPDS*-sgRNA were used to analyze mobile sgRNA efficiency, and the top leaves at 70 DPI were used for gDNA extraction. gDNA was diluted to 50 ng/μL for analysis. Total RNA was extracted from infiltrated cotyledons, systemic leaves and *SlPDS* E_1_ seedlings respectively. At least three replicate samples were collected and pooled at 2-day intervals from 4 to 10 DPI to ensure the repeatability of the RNA extraction in PVX-inoculated samples. Total RNA was extracted using a TaKaRaMiniBEST Plant RNA Extraction kit (Takara Bio, Kusatsu, Japan). An AccuPower RT PreMix kit (Bioneer, Daejeon, Korea) with oligo (dT) primers was used to synthesize first-strand cDNA from 800 ng total RNA. The resulting cDNA was used for qRT-PCR and RT-PCR.

### Analysis of transgene expression in PVX-inoculated cotyledons

To examine the expression of the transgene from the PVX vector, qPCR was performed on a light Cycler 480 system (Roche, Basel, Switzerland) using a modified SYTO9 stain method [62]. The qRT-PCR primer sequences are listed in Table 3. Three technical replicate reactions were performed. A housekeeping gene, *SlActin*, was used as a control and expression levels analysis follow the delta Ct method.

**Table 3:** List of primers used for RT-qPCR analysis and transgene integration in PVX-mediated GE

**Gene**	**Primer sequence (5′ to 3′)**
*RdRp*	F: GCAGCCTTTTACCAGCAGAC R: ACATGGCTCCATCCTGAGAC
*Cas9*	F: ATGAGCACCACCAGGATCTC R: TCGGTTCCATCCATCTTCTC
*CP*	F: ACACAGCCCATAGGGTCAAC R: GCCTCAATCTTGCTGAGGTC
*SlActin*	F: TGGTTACTCGTTCACCACCT R: AGGACACCGGAAACGCTCA

### Immunoblot assay of SpCas9 in *Cas9* transgenic MT and PVX-inoculated cotyledons

The cotyledons from CMT T_3_ homozygous lines and PVX-inoculated MT plants were collected every other day from 3 to 5 DPI and 4 to 10 DPI to detect Cas9, respectively. Leaf samples were frozen in liquid nitrogen and ground to a fine powder using a pestle and mortar, before mixing the powder with 80 μL protein extraction buffer as described with some modifications [63]. After 10 s of sonication, the samples were boiled at 98°C for 10 minutes, and 30 μL of supernatant was extracted and used in the experiment after centrifugation at 4°C for 2 minutes at 13000 g. The proteins were separated by 8% SDS-PAGE at 120 V for 2 hours. Following electrophoresis, one replicate was transferred to an Amersham Hybond–P membrane (GE Healthcare, Chicago, USA) following the manufacturer’s instructions. The other replicate was stained with D-plus Protein gel Staining Solution (Dongin Biotech, Seoul, Korea). The membrane was blocked using Tris-buffered saline with Tween 20 (TBST) containing 5% (w/v) BSA for 1 hour at room temperature. The blocked membranes were incubated in HRP-conjugated anti-CRISPR/Cas9 antibody (7A9-3A3, ab202580; Abcam) diluted 1:2000 in TBST containing 5% (v/v) BSA at room temperature for 1 hour. The membranes were washed three times in 1× TBST with shaking (15 minutes each time). The signals were detected using Clarity Western ECL substrate (Bio-Rad) and Fusion FX (Vilber Lourmat, Collegien, France) following the manufacturer’s instructions.

### CAPS analysis for mutation detection

For the CAPS analysis, a pair of gene-specific primers was designed to amplify the target site of *SlPDS*, *StPDS,* and *SmPDS*. PCR amplification of the targeted genomic region was performed using specific primers listed in Table S2. Purification of each PCR amplicon was conducted with AMPure XP beads (Beckman Coulter, Brea CA, USA). Cleavage of the purified PCR products was performed at 37°C for 8 hours in a mixture containing 500 ng PCR amplicon, 2 μL 10× CutSmart buffer (NEB, Beverly, MA), and 1 μL *Hinf*I (PVX-*SlPDS*) (NEB, Beverly, MA), *Sac*l-HF (PVX-*St&mPDS1*) (NEB, Beverly, MA), *Bbs*l-HF (PVX-*StPDS2*) (NEB, Beverly, MA), or *Dra*I (PVX-*StPDS3* and PVX-*SmPDS2*) (NEB, Beverly, MA) in a final volume of 20 μL. The products were separated by electrophoresis at 135 V for 30 minutes on a 2% (w/v) agarose gel. Band intensity was quantified using ImageJ software (NIH, Bethesda, MD, USA).

### Heat treatment (HT) of samples inoculated with VIGE constructs

Tomato plants inoculated with TRV-*SlPDS*-sgRNA were used to evaluate the effects of HT on editing efficiency. To optimize the timing of HT, explants from the CMT plants inoculated with TRV-*SlPDS*-sgRNA were subjected to a 24-hour HT at 37°C from 2 to 5 DPI. In another test, HT was conducted at 3 DPI for 12 or 24 hours, with 12 hours of HT at 3 DPI being selected to regenerate edited shoots. To compare the mutation rate in regenerated shoots with or without HT (NT), each shoot was evaluated by CAPS analysis (Table S2). Similarly, MT plants inoculated with PVX-*SlPDS* were heat-treated to examine the effects of HT on editing efficiency. The experiment was conducted twice, and each sample was subjected to a 24-hour HT at 37°C. During the first experiment, HT was performed every other day starting at 3 DPI. ELISA and CAPS analysis were conducted to analyze PVX virus accumulation and editing efficiency in heat-treated samples. Based on the results of the first experiment, HT was performed at 7 and 9 DPI, which were the most effective times. In the second experiment, explants were surface-sterilized and placed in tissue culture medium before being subjected to HT. The experiment was conducted on potato and eggplant inoculated with PVX-gRNA (*St&mPDS1*, *StPDS2*, *StPDS3*, *SmPDS2*) to examine editing efficiency induced by HT. HT was performed at 9 DPI, which was the most effective in tomato. Three replicate reactions were performed.

### Analysis of editing efficiency and mutation pattern by deep sequencing

A 402-bp amplicon, including the target site, was used for deep sequencing. Deep sequencing primers were synthesized at Macrogen (Seoul, Korea). Table S3 lists the primers with multiplexing indices and sequencing adaptors required for PCR amplification of sgRNA target regions and deep sequencing. Target regions were amplified using a QIAGEN Multiplex PCR kit (QIAGEN, Hilden, Germany). The resulting amplicons were separated on a 2% (w/v) agarose gel. The PCR products were recovered using a LaboPass PCR Clean-up kit (Cosmo Genetech, Seoul, Korea). Pooled PCR samples were sequenced on an Illumina Miseq platform at NICEM (Seoul National University, Seoul, Korea). Raw paired-end reads were analyzed, as described by Venkatesh et al. (2022) [33] with some modifications. Insertions and deletions (InDels) were estimated within and around the target region. GE efficiency was calculated as the ratio of DNA sequence reads with non-homologous DNA end joining-induced InDels over the total number of reads.

### Generation of edited plants through the VIGE system in tomato

VIGE tissue culture was performed as described by Yoon et al. (2020) with some modifications. Cotyledons from seedlings inoculated with TRV-*SlPDS*-sgRNA at 2 DPI or with PVX-*SlPDS* at 4 DPI were surface-sterilized with 70% (v/v) ethanol for 1 minute and 0.4% (w/v) NaOCl with a drop of Tween 20 for 10 minutes, and then rinsed five times with sterilized water. After surface sterilization, cotyledons were used for tissue culture by transferring the explants to the growth media listed in Table 2.

### Evaluation of transgene integration in edited plants from the PVX-mediated GE system

To verify transgene integration in the edited plants, transgene-specific primers were designed based on the *Cas9* and *CP* sequences of the PVX vector and synthesized by Macrogen (Seoul, Korea). PCR amplification of the targeted region was performed using specific primers listed in Table S2. The PCR products were separated by electrophoresis on a 2% (w/v) agarose gel at 135 V for 40 minutes.

## Supplementary Material

Web_Material_uhad233

## Data Availability

All data used in this study are included in these manuscripts and supplementary data.
