# Supplementary material for: Development of virus-induced genome editing methods in Solanaceous crops
Source: Hortic Res. 2023 Nov 17;11(1):uhad233. doi: 10.1093/hr/uhad233 (PMC10782499; doi:10.1093/hr/uhad233)
Supplement: Web_Material_uhad233 [file web_material_uhad233.zip › 231015_VIGE-in-solanaceous-crops-MS_Horticulture-Research_Supplementary Data_Final.docx]

# **Supplementary tables**

## **Table S1. Primers used for generating transgenic tomato lines expressing *Cas9*.**

| Primer | Sequence (5’ to 3’) | Purpose |
| --- | --- | --- |
| Cas9_pHSE-F | ATCCAATCTTCGGCAACAT | *Cas9* amplification |
| Cas9_pHSE-R | TTATCCAGGTCATCGTCGTAT |  |
| pHSE401-vector-F | AAAATCCAGTACTAAAATCCAGATCC | pHSE401 vector amplification |
| pHSE401-vector-R | CTCCAATTCACTGTTCCTTGC |  |
| SlActin-F | AGTGGTGGTACTACCATGTTCCCA | Tomato *Actin* amplification |
| SlActin-R | GAGGGAAGCCAAGATAGAGCCTCCA |  |

## **Table S2. Primers used in this study.**

| Primer | Sequence (5’ to 3’) | Purpose |
| --- | --- | --- |
| SlPDSMfeI-F | CGAGCAATTGCTGTTAACTTGAGAGTC | TRV-*SlPDS*-sgRNA vector construction |
| SlPDSXmaI-R | CATGCCCGGGAATTCTGAGGAGAAGAG |  |
| SlPDSStuI-R | TACCAGGCCTGCACCGACTCGGT |  |
| FtsgRNAStuI-F | CGCCAGGCCTTAGTCTATAAATATAAGAGAC |  |
| FtsgRNAXmaI-R | ATATCCCGGGCTTTGGCCATAAGTAA |  |
| tRNAIlesgRNAStuI-F | GGCAAGGCCTGCTCCCGTAGCTCAGTTGGT |  |
| tRNAIlesgRNAXmaI-R | TAATCCCGGGTGCTTCCGGCGGGGCT |  |
| TRV_seq_F | CTGTTTGAGGGAAAAGTAG | TRV sequence confirmation |
| TRV_seq_R | CAAAAGACTTACCGATCAATC |  |
| TRV2.CP-F | TCCTGCTGACTTGATGGACGAT | TRV coat protein amplification |
| TRV2.CP-R | CCACTGCATTCTTCTCATCAGCTC |  |
| SIPDS-MluI-F1 | TTAACTTGAGAGTCCAGTTTTAGAGCTAGAAATA | PVX vector construction |
| SlPDS-MluI-F2 | ATGCACGCGTCTGTTAACTTGAGAGTCCA |  |
| StPDS-gRNA1_F1 | ATTCGGGAGCTCGTGGGTTTTAGAGCTAGAAATA |  |
| StPDS-gRNA1_F2 | ATGCACGCGTCAGCTTATCTTTGGAGCTCG |  |
| StPDS-gRNA2_F1 | TCGAGGTCGTCTTCTTGTTTTAGAGCTAGAAATA |  |
| StPDS-gRNA2_F2 | ATGCACGCGTGAGCTCGAGGTCGTCTTCTT |  |
| StPDS-gRNA3_F1 | GACTTGGGGCCTTTAAGTTTTAGAGCTAGAAATA |  |
| StPDS-gRNA3_F2 | ATGCACGCGTTAAGGACTTGGGGCCTTTAA |  |
| SmPDS-gRNA1_F1 | TTATCTTTGGAGCTCGGTTTTAGAGCTAGAAATA |  |
| SmPDS-gRNA1_F2 | ATGCACGCGTCAGCTTATCTTTGGAGCTCG |  |
| SmPDS-gRNA2_F1 | CCAAACCTTTAAAGGCGTTTTAGAGCTAGAAATA |  |
| SmPDS-gRNA2_F2 | ATGCACGCGTCAAACCAAACCTTTAAAGGC |  |
| SIPDS- gRNA1_F1 | TTAACTTGAGAGTCCAGTTTTAGAGCTAGAAATA |  |
| SlPDS- gRNA1_F2 | ATGCACGCGTCTGTTAACTTGAGAGTCCA |  |
| PVX-SalI-R1 | CGGCGGTCGACTGGGTCTAGAAAAAAAGCA |  |
| PVX-SalI-R2 | CGTTCATCGGCGGTCGACTGGGTCTAG |  |
| SmPDS-MD-F | CTGTAAGAATGAGACGGTAAAGC | CAPS analysis for *SmPDS* mutation detection |
| SmPDS-MD-R | ACCTGCACCAGCAATAACAA |  |
| StPDS-MD-F | CTACTTGATTTTGTGCACAG | CAPS analysis for *StPDS* mutation detection |
| StPDS-MD-R | GAATGCTACAAATATCACCTGCAC |  |
| SlPDS-T-F | AGCATTCGGTATCTTTTTCTGGGTAACT | CAPS analysis for *SlPDS* mutation detection in PVX mediated GE system |
| SlPDS-T-R | CCAGCAAAACATAACGAATTCCTTTGCA |  |
| SlPDS-gRNA-F | TCTGGGTAACTGCCAAACCA | *SlPDS* sequencing for mutation detection |
| SlPDS-gRNA-R | TTGTCCAGCTCTGGTCTTGG |  |
| SlPDS-PCR-RE-F | TTACTGTGAAATATCCTTATGGCAGGT | CAPS analysis for *SlPDS* mutation detection |
| SlPDS-PCR-RE-R | CATAACGAATTCCTTTGCAAGCAAC |  |
| PVX-Cas9-F | TGGTTTCGATTCTCCTACCG | Analysis of transgene integration from PVX-mediated GE system |
| PVX-Cas9-R | ATCAGCCCTTGAATCACCAC |  |
| PVX-CP-F | ACCAGCTAGCACAACACAGC |  |
| PVX-CP-R | GTTATGGTGGTGGTAGAGTGA |  |
| SlPDS-integration-F | AGCATTCGGTATCTTTTTCTGGGTAACT |  |
| SlPDS-integration-R | CCAGCAAAACATAACGAATTCCTTTGCA |  |

## **Table S3. Primer sequences used for deep sequencing.**

| Gene | Primer sequence (5′ to 3′) | Pool No |
| --- | --- | --- |
| SlPDS_Miseq_1PCR_1F | TCGTCGGCAGCGTCAGATGTGTATAAGAGACAGNNNNN**AACAC**CATAAATGCTTCTCAACATAAAT | 1 |
| SlPDS_Miseq_1PCR_1F-1 | TCGTCGGCAGCGTCAGATGTGTATAAGAGACAGNNNNN**AACAC**NCATAAATGCTTCTCAACATAAAT |  |
| SlPDS_Miseq_1PCR_1F-2 | TCGTCGGCAGCGTCAGATGTGTATAAGAGACAGNNNNN**AACAC**NNCATAAATGCTTCTCAACATAAAT |  |
| SlPDS_Miseq_1PCR_1F-3 | TCGTCGGCAGCGTCAGATGTGTATAAGAGACAGNNNNN**AACAC**NNNCATAAATGCTTCTCAACATAAAT |  |
| SlPDS_Miseq_1PCR_2F | TCGTCGGCAGCGTCAGATGTGTATAAGAGACAGNNNNN**ACTAT**CATAAATGCTTCTCAACATAAAT | 2 |
| SlPDS_Miseq_1PCR_2F-1 | TCGTCGGCAGCGTCAGATGTGTATAAGAGACAGNNNNN**ACTAT**NCATAAATGCTTCTCAACATAAAT |  |
| SlPDS_Miseq_1PCR_2F-2 | TCGTCGGCAGCGTCAGATGTGTATAAGAGACAGNNNNN**ACTAT**NNCATAAATGCTTCTCAACATAAAT |  |
| SlPDS_Miseq_1PCR_2F-3 | TCGTCGGCAGCGTCAGATGTGTATAAGAGACAGNNNNN**ACTAT**NNNCATAAATGCTTCTCAACATAAAT |  |
| SlPDS_Miseq_1PCR_3F | TCGTCGGCAGCGTCAGATGTGTATAAGAGACAGNNNNN**ATCACG**CATAAATGCTTCTCAACATAAAT | 3 |
| SlPDS_Miseq_1PCR_3F-1 | TCGTCGGCAGCGTCAGATGTGTATAAGAGACAGNNNNN**ATCACG**NCATAAATGCTTCTCAACATAAAT |  |
| SlPDS_Miseq_1PCR_3F-2 | TCGTCGGCAGCGTCAGATGTGTATAAGAGACAGNNNNN**ATCACG**NNCATAAATGCTTCTCAACATAAAT |  |
| SlPDS_Miseq_1PCR_3F-3 | TCGTCGGCAGCGTCAGATGTGTATAAGAGACAGNNNNN**ATCACG**NNNCATAAATGCTTCTCAACATAAAT |  |
| SlPDS_Miseq_1PCR_1R | GTCTCGTGGGCTCGGAGATGTGTATAAGAGACAGGCCTTGAGATAATAATTCAAGTC | Common reverse |
| SlPDS_Miseq_1PCR_1R-1 | GTCTCGTGGGCTCGGAGATGTGTATAAGAGACAGNGCCTTGAGATAATAATTCAAGTC |  |
| SlPDS_Miseq_1PCR_1R-2 | GTCTCGTGGGCTCGGAGATGTGTATAAGAGACAGNNGCCTTGAGATAATAATTCAAGTC |  |
| SlPDS_Miseq_1PCR_1R-3 | GTCTCGTGGGCTCGGAGATGTGTATAAGAGACAGNNNGCCTTGAGATAATAATTCAAGTC |  |

Red fonts indicate pool specific barcode

# **Supplementary figures**


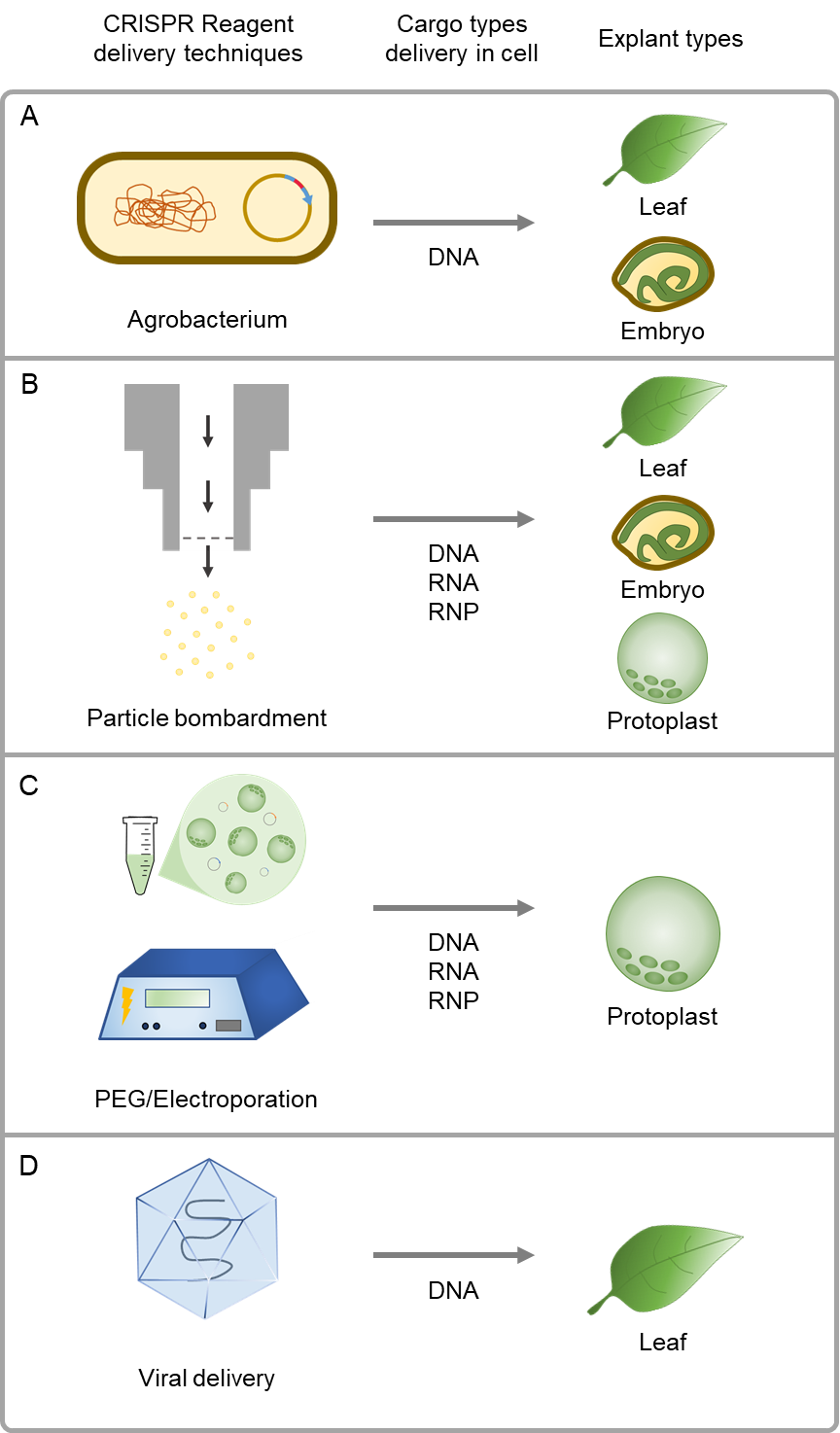


**Fig. S1. An overview of various methods used to deliver GE reagents in plant cells.**


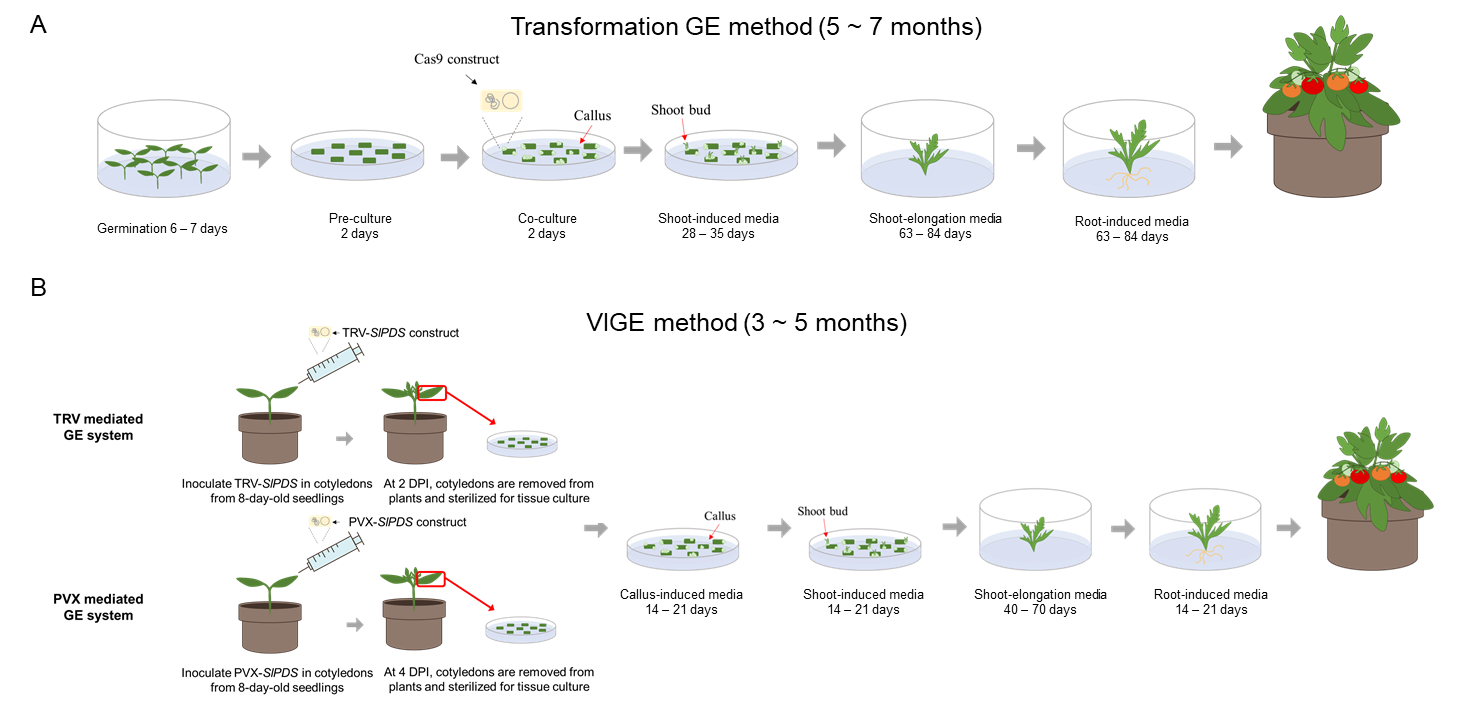


### **Fig. S2. Diagram of the conventional *Agrobacterium*-mediated transformation method and VIGE method.**

**A** Diagrams of the conventional *Agrobacterium*-mediated transformation method. **B** Diagrams of the VIGE method.

**
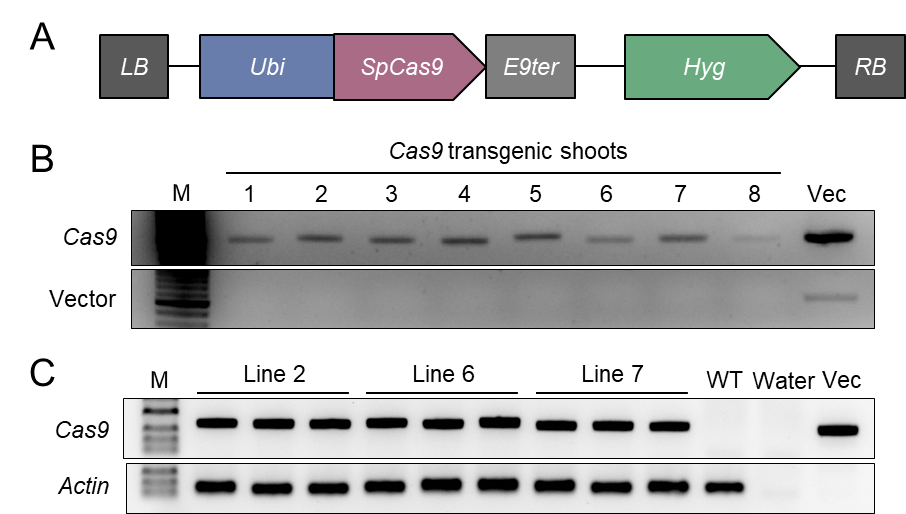
**

**Fig. S3. Generation and confirmation of *Cas9* transgenic tomato plants.**

**A** Diagram of the pHSE401-*Cas9* vector, with *Cas9* driven by the *Ubiquitin* promoter. *LB*, Left border; *Ubi*, Maize *Ubiquitin* promoter; *SpCas9*, *Streptococcus pyogenes Cas9*; *E9ter*, pea (*Pisum sativum*) RuBisCO small subunit E9 terminator; *Hyg*, hygromycin resistance gene; *RB*, Right border. **B** PCR genotyping for *Cas9* in T_0_ shoots. Vector-specific PCR primers were used to test for *Agrobacterium* contamination. **C** Confirmation of *Cas9* expression, as determined by RT-PCR in T_1_ plants of each transgenic line. WT, wild-type plant; Vec, pHSE401-*Cas9* vector.


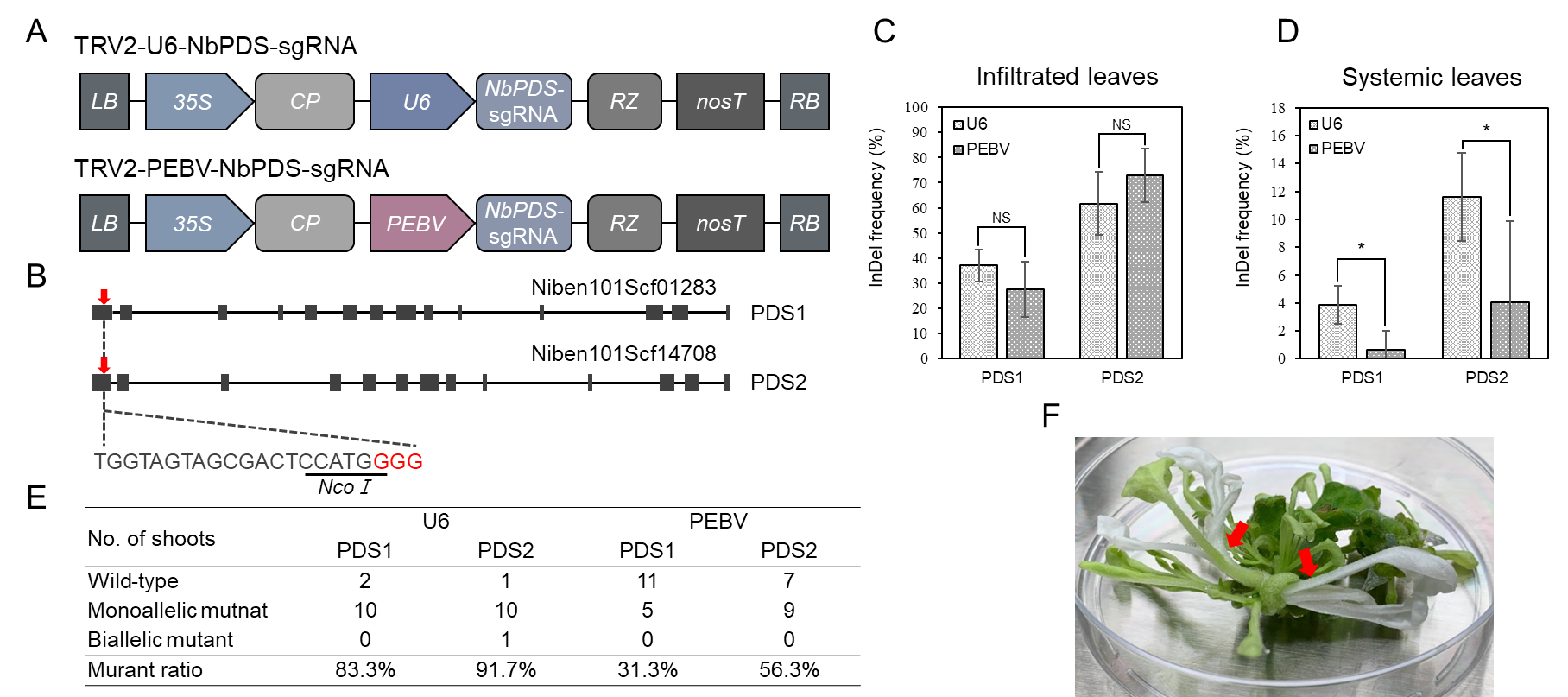


**Fig. S4. Comparison of editing efficiency by sgRNA promoter in TRV-mediated GE.**

**A** Diagrams of TRV-sgRNA constructs with different promoters. *LB, Left border; 35S, Cauliflower mosaic virus 35S promoter; CP, coat protein; U6, AtU6-26 promoter; RZ, terminating ribozyme; nosT, nopaline synthase terminator; RB, Right border*. **B** NbPDS homologs and sgRNA target sites. Red arrows indicates sgRNA target sites. The restriction enzyme, *NcoⅠ*, used for mutation detection, is underlined in the sgRNA target sequence. Red fonts indicate the PAM site**. C, D** Editing efficiencies of *U6-26* and PEBV promoters in the infiltrated (C) and systemic leaves (D). Error bars represent standard deviation (SD) of mean. **E** Mutant ratio under *U6-26* and PEBV promoters in regenerated shoots. **F** Photobleached shoots regenerated from TRV-U6-NbPDS-sgRNA infiltrated plant.

**
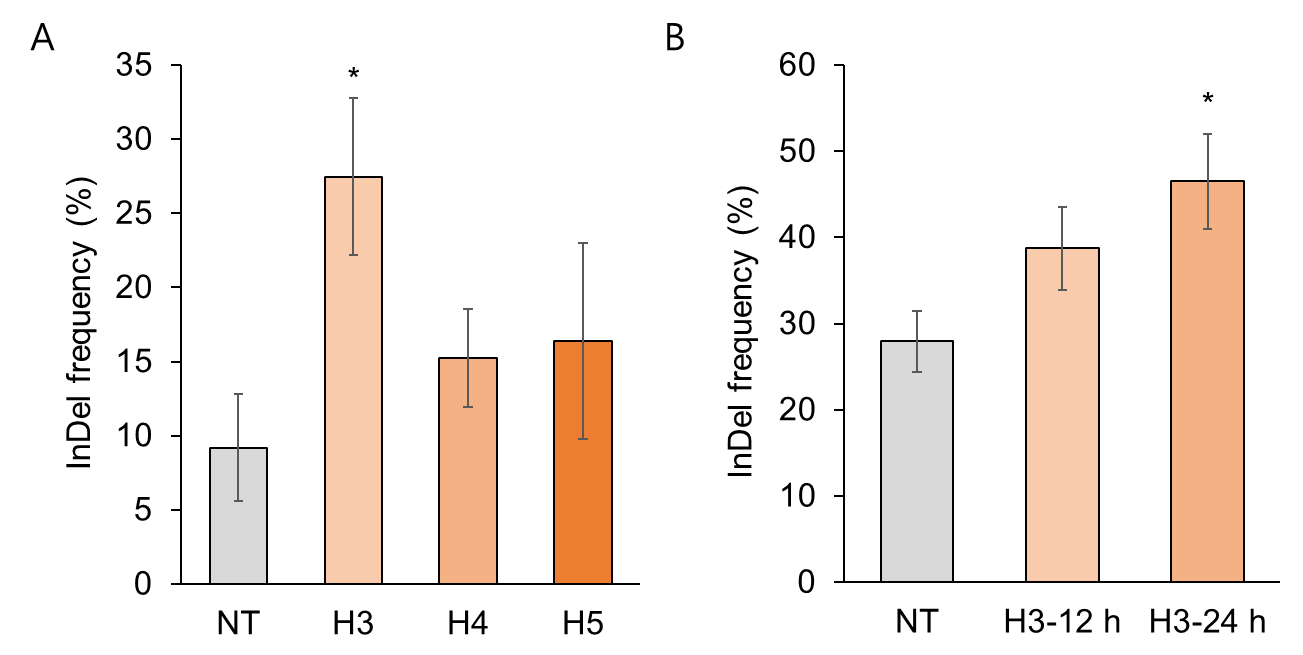
**

**Fig. S5.** **Effects of heat treatment on TRV-mediated GE of *SlPDS* in tomato analyzed by** **DECORD tool.**

**A** InDel frequency after a 24-h HT in MT inoculated with TRV-*SlPDS*-sgRNA. The InDel frequency was analyzed by DECORD tool. Five biological replicates were used for each treatment. **B** InDel frequency after a 12-h or 24-h HT at 3 DPI. At least eight biological replicates were used for each treatment. NT, Normal condition (23℃); H3, 37℃ for 24 h starting at 3 DPI; H4, 37℃ for 24 h starting at 4 DPI; H5, 37℃ for 24 h starting at 5 DPI; H3-12 h, 37℃ for 12 h starting at 3 DPI; H3-24 h, 37℃ for 24 h starting at 3 DPI. The significant difference was determined using a t-test: *P<0.05.


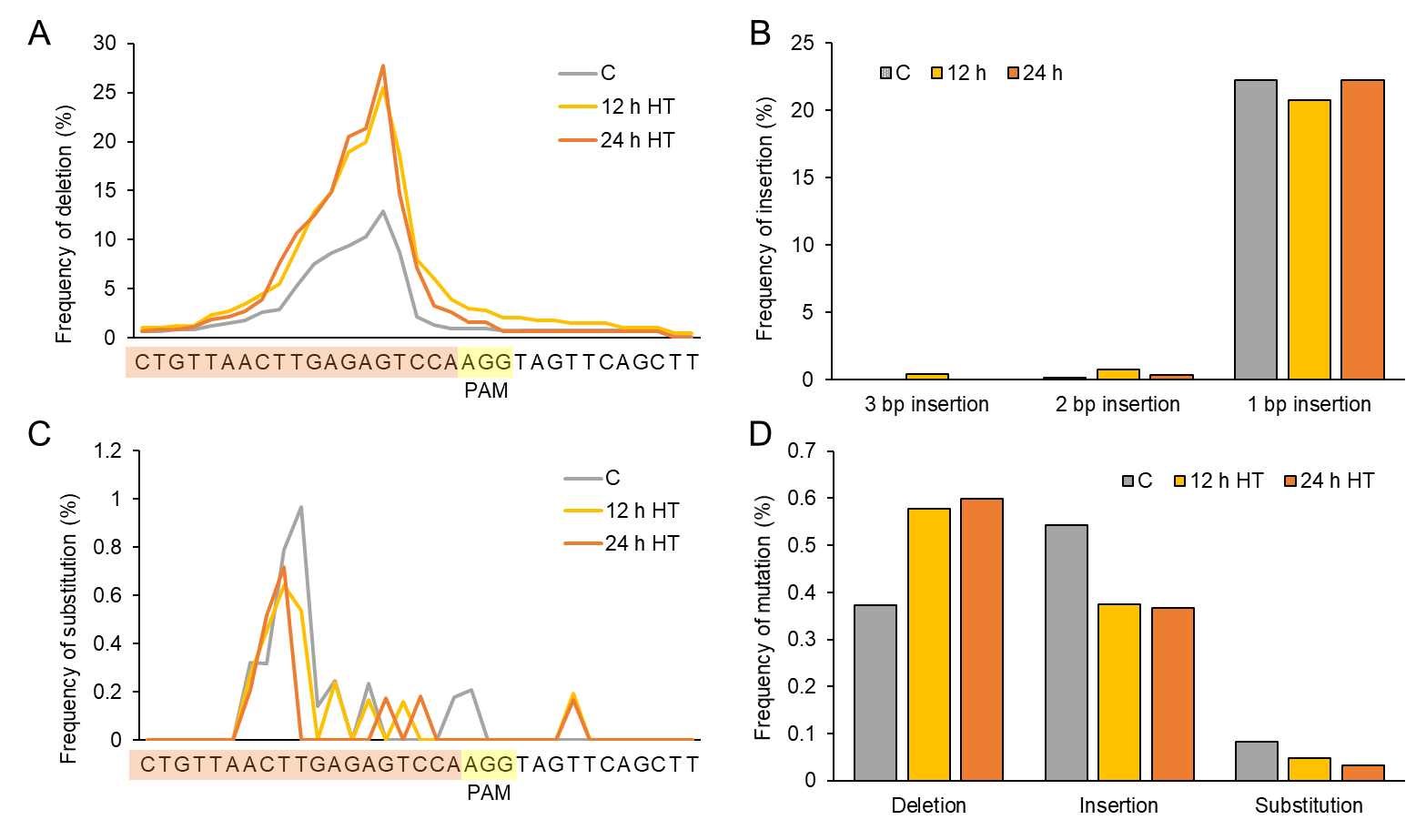


**Fig. S6. Effect of HT on TRV-mediated GE in tomato.**

To investigate the patterns of mutations induced by the TRV-mediated GE system, the frequency of InDels and substitutions was analyzed by deep sequencing. **A** Frequency of sequence changes across the site of the *SlPDS* target region. **B** Frequency of different insertion lengths along the *SlPDS* target region. **C** Frequency of substitutions along the *SlPDS* target region. **D** Summary of sequence variations identified by deep sequencing. The target region is highlighted in orange; the protospacer adjacent motif (PAM) is highlighted in yellow. C, Normal condition (23°C); 12 h HT, 37°C for 12 h starting at 3 DPI; 24 h HT, 37°C for 24 h starting at 3 DPI.


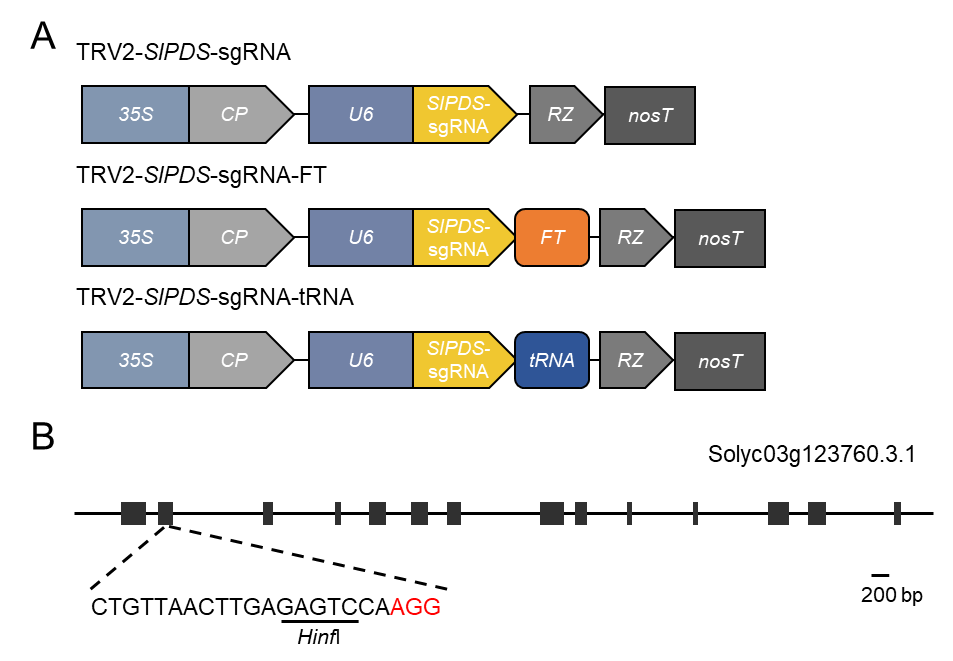


**Fig. S7. Mobile TRV2-sgRNA constructs targeting *SlPDS* in tomato.**

**A** Diagrams of the mobile TRV2-sgRNA. sgRNAs were modified by adding mobile mRNA sequences (*FT* and tRNA-Ile) to improve editing efficiency. *35S*, Cauliflower mosaic virus (CaMV) *35S* promoter; *CP*, coat protein gene; *U6*, *U6-26* promoter; *FT*, *FLOWERING LOCUS T*; *tRNA-Ile*, tRNA Isoleucine; *RZ*, terminating ribozyme; *nosT*, nopaline synthase terminator. **B** Diagram of the *SlPDS* locus and sgRNA target site.

**
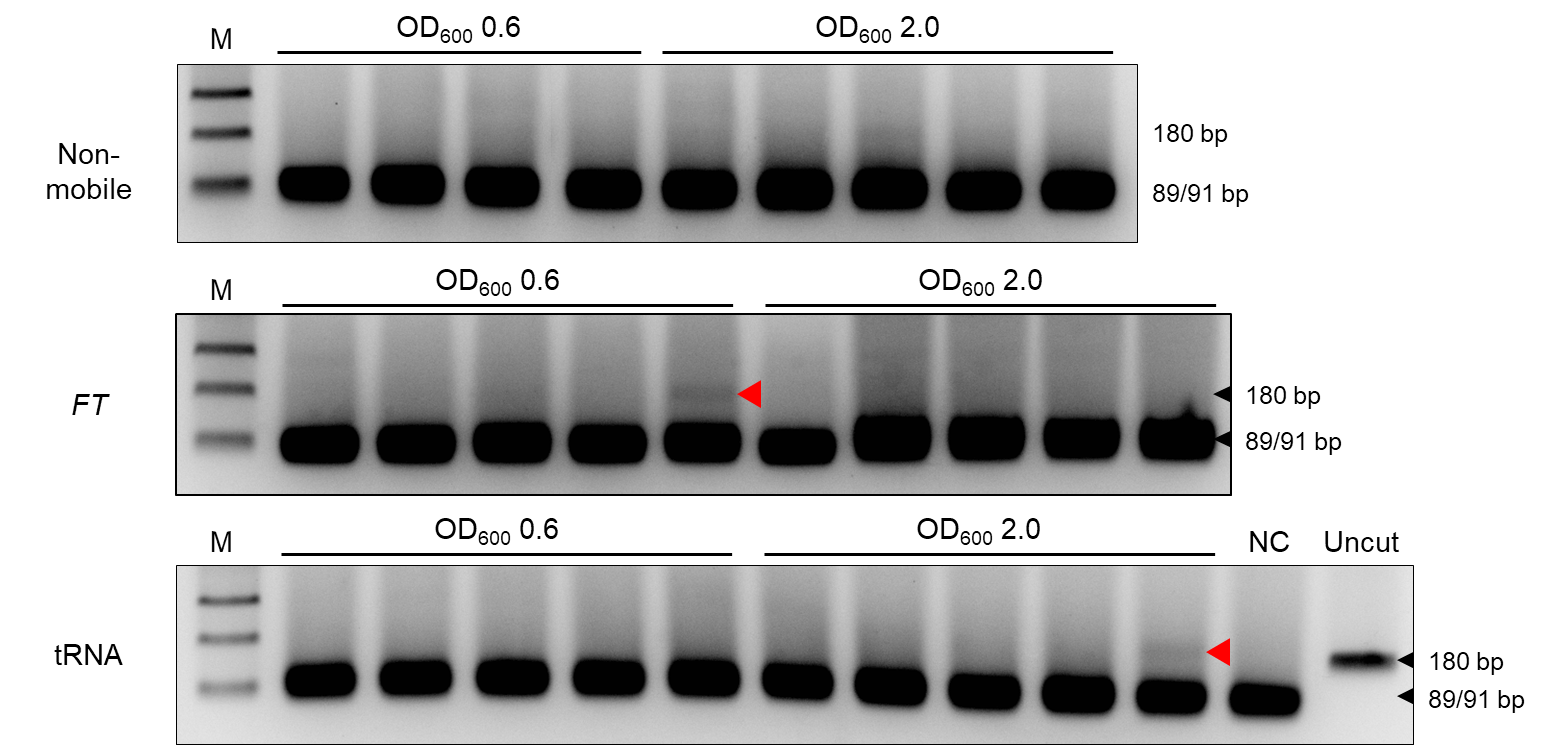
**

**Fig. S8. Mutation detection at *SlPDS* in systemic leaves from tomato plants inoculated with TRV mobile sgRNA constructs.**

Non-mobile sgRNA and mobile sgRNA (FT and tRNA) were infiltrated into the cotyledons of MT seedlings, and after 70 DPI, the three most upper leaves were pooled for mutation detection. CAPS analysis was performed using *Hinf*I and gDNA extracted from leaves. The red arrows indicate the uncut band due to mutations in the *SlPDS* target region.


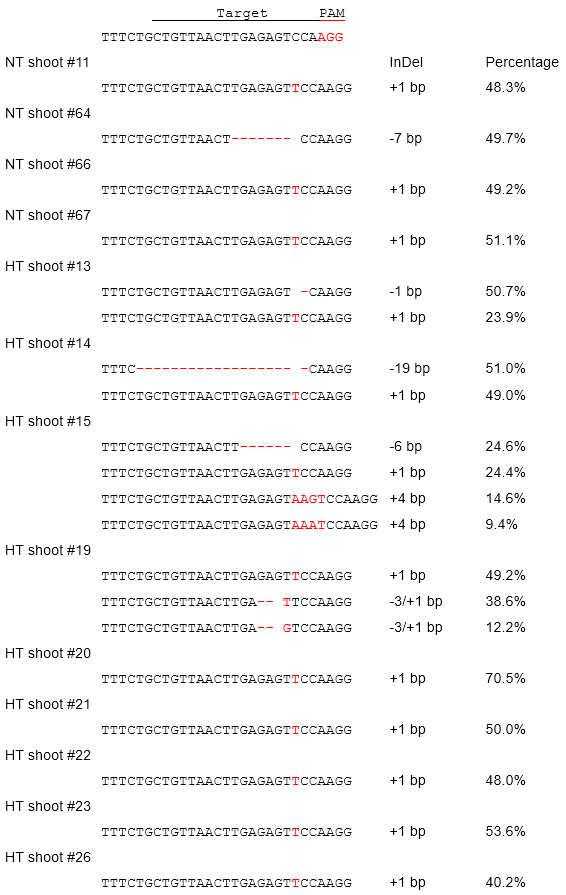

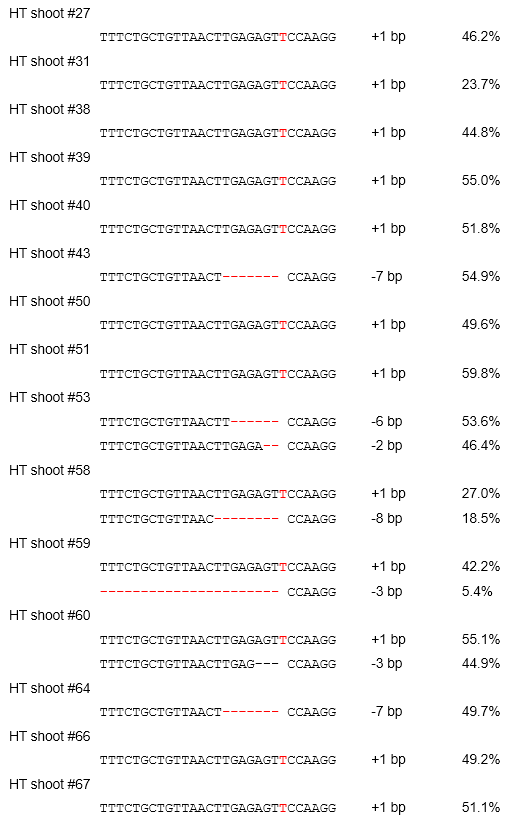


### **Fig. S9. Analysis of** ***SlPDS* sequences in regenerated E_0_ mutant tomato plants developed through TRV-mediated GE.**

The *SlPDS* target region was amplified from 28 regenerated E_0_ shoots, and the inference of DECODR tool was used to detect mutations. The type of mutations and the percentage of each mutation are shown on the right side. NT shoot, Shoot from normal condition (23℃); HS shoot, Shoot from 12h HT at 37℃.

**
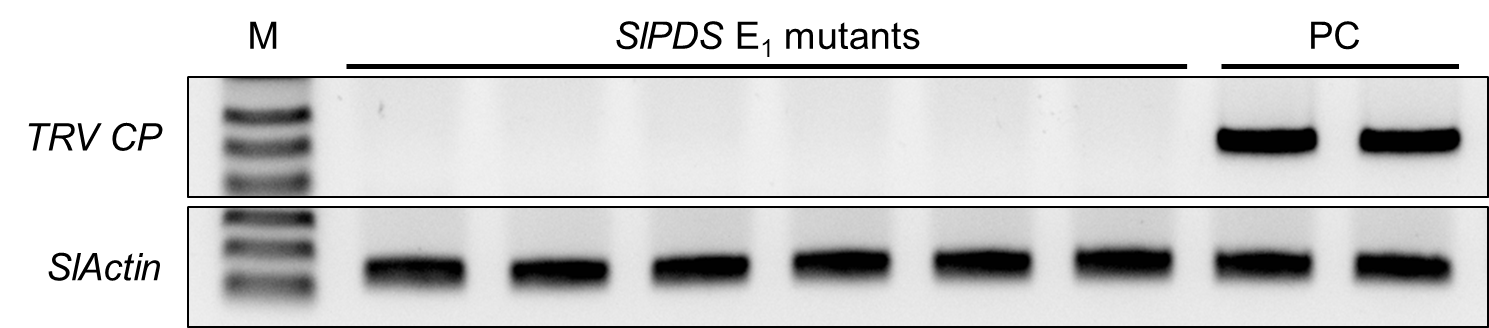
**

**Fig. S10. TRV detection in the E_1_ progenies of TRV-mediated GE.**

To check transmission of TRV in the next generation, total RNA extracted from *SlPDS* E_1_ progenies was used for RT-PCR. TRV coat protein was amplified with the primer listed in Table S2. *SlActin* was amplified as a control with the primer listed in Table S1. *TRV CP*, TRV coat protein; PC, TRV infiltrated leaves at 6 DPI for positive control.


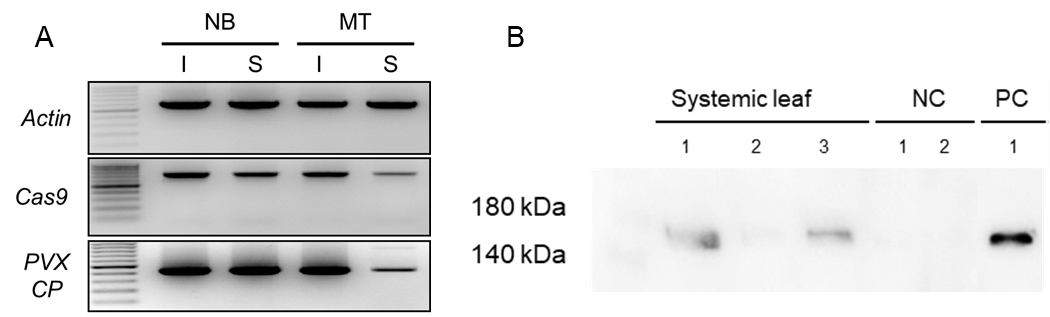


**Fig. S11. Confirmation of foreign gene expression in *S. lycopersicum* cv. MT systemic leaf inoculated with the PVX-SlPDS vector.**

*Cas9* expression was detected in the systemic leaves of *N. benthamiana* and MT plants that were inoculated with PVX-sgRNA at 7 DPI. *Actin* was used as an internal reference. CP was used to confirm PVX infection. *CP*, PVX coat protein gene; NB, *N. benthamiana*; MT, Micro-Tom; I, inoculated leaf; S, systemic leaf. **B** Immunoblot analysis of MT systemic leaf inoculated with the PVX-SlPDS construct. Cas9 was detected using Cas9-specific antibodies. Samples were collected from MT inoculated with PVX-SlPDS from 14 DPI. NC, negative control (MT infiltrated with infiltration buffer); PC, positive control (Cas9-positive transgenic MT).


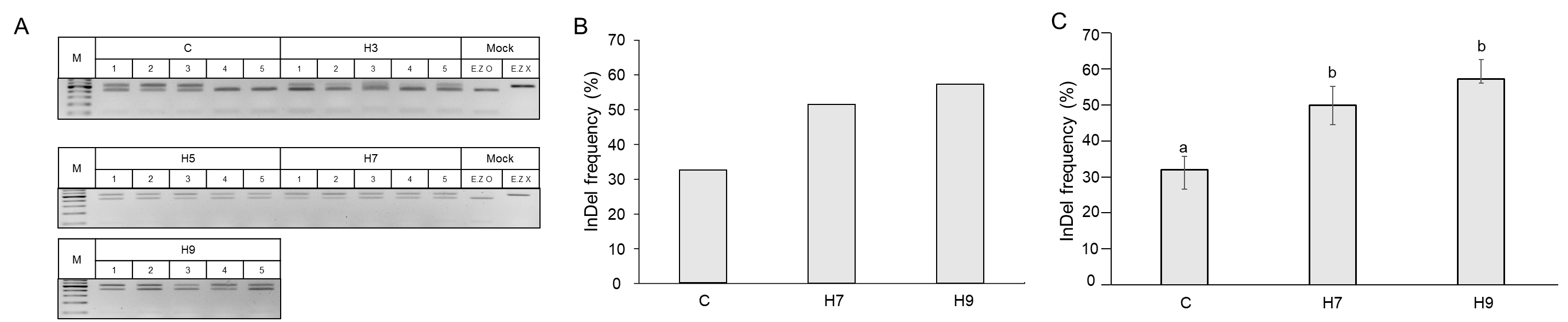


**Fig. S12. Effect of HT on PVX-mediated GE of *SlPDS* in tomato.**

**A** CAPS analysis of cotyledon inoculated with PVX-*SlPDS*, performed at 10 DPI. Seedlings were subjected to HT every 2 days, starting from 3 DPI until 9 DPI: WT/mock: 423 bp and 102 bp; Mutant: 525 bp. Mock, infiltrated with infiltration buffer; C, Normal condition (23℃); H3, 37℃ for 24 h starting at 3 DPI; H5, 37℃ for 24 h starting at 5 DPI; H7, 37℃ for 24 h starting at 7 DPI; H9, 37℃ for 24 h starting at 9 DPI; E.Z O, wild-type PCR product incubated with restriction enzyme; E.Z X, wild-type PCR product. **B** InDel frequency after a 24-h HT in MT inoculated with PVX-*SlPDS*. A CAPS analysis was performed to estimate InDel frequency. DNA band intensities in the gel image were quantified by ImageJ software. **C** InDel frequency analyzed by DECORD tool after a 24-h HT in MT inoculated with PVX-*SlPDS*. Three biological replicates were used for each treatment. Values are means ± SD. Different lowercase letters indicate a significant difference according to Tukey’s test (*P* < 0.05).


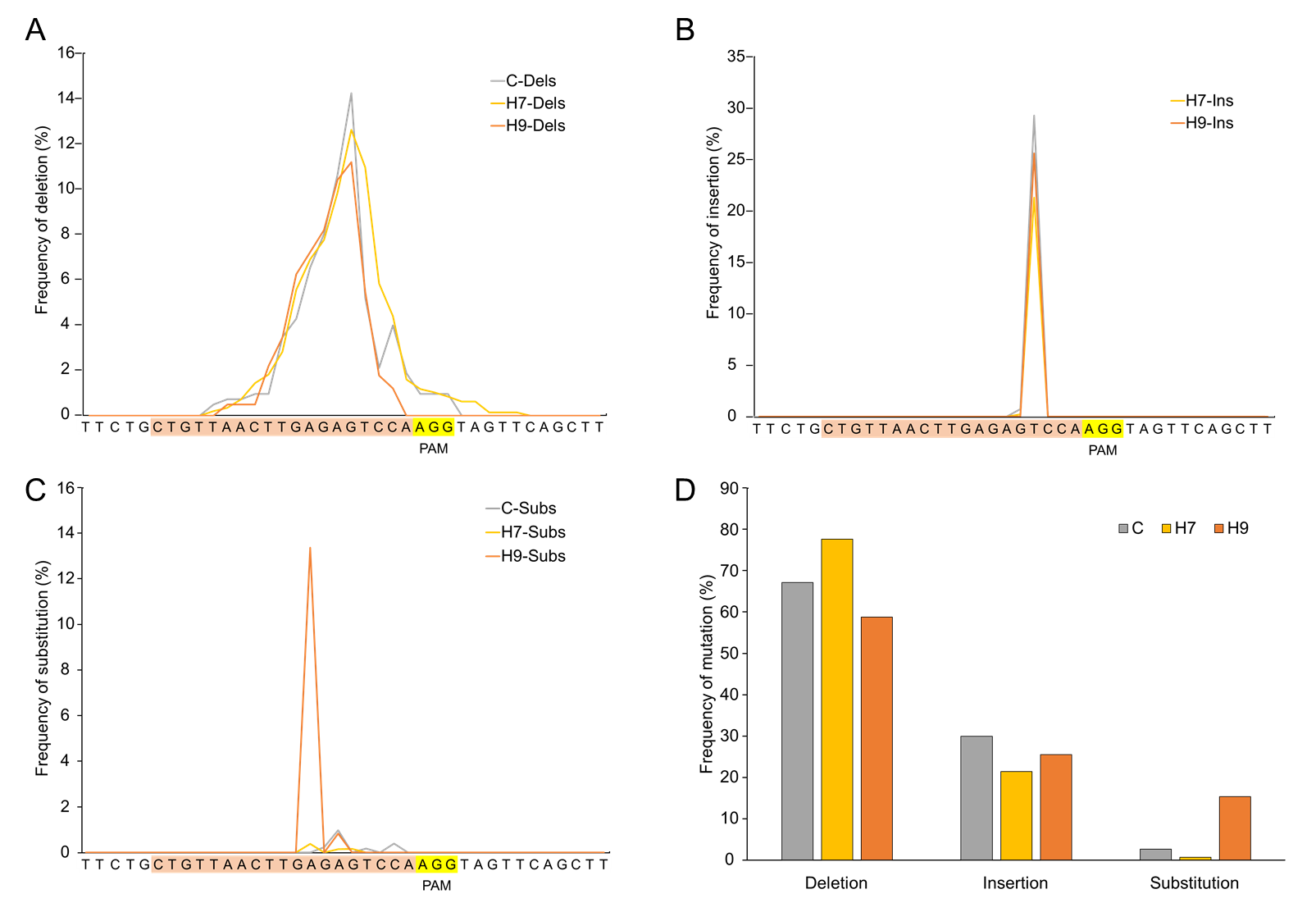


**Fig. S13. Effect of HT on PVX-mediated GE in tomato.**

To investigate the specificities of the targeted GE at the *SlPDS* target site, deletions (Dels), insertions (Ins), and substitutions (Subs) were analyzed by deep sequencing. **A** Frequency of different deletions along the target region in MT cotyledons inoculated with PVX-*SlPDS*. **B** Frequency of different insertions along the target region in MT cotyledons inoculated with PVX-*SlPDS*. **C** Frequency of substitutions along the target region in MT cotyledons inoculated with PVX-*SlPDS*. **D** Summary of sequence variation identified by deep sequencing. The target region is highlighted in orange; the PAM is highlighted in yellow; red font indicates the *Hinf*I restriction site. C, Normal condition, (23°C); H7, 37°C for 24 h starting at 7 DPI; H9, 37°C for 24 h starting at 9 DPI.

**
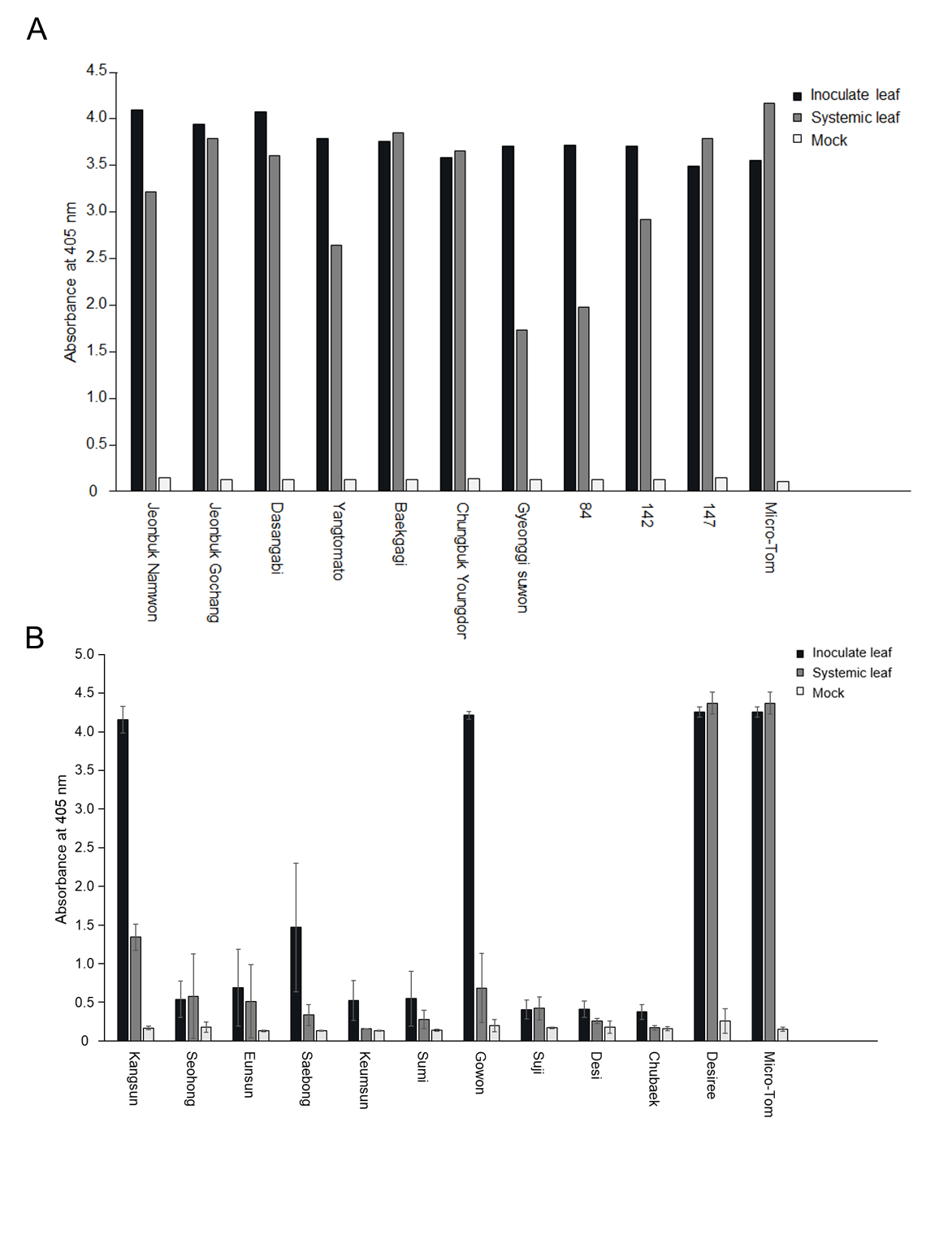
**

**Fig. S14. Analysis of PVX-CP accumulation in potato and eggplant.**

Eleven potato and 10 eggplant cultivars were used for a PVX susceptibility test. **A** PVX-CP accumulation in inoculated and systemic leaves of potato plants inoculated with PVX-GFP, as determined by ELISA at 10 DPI. **B** PVX-CP accumulation in inoculated and systemic leaves of eggplant plants inoculated with PVX-GFP, as determined by ELISA at 10 DPI. Micro-Tom was used as a positive control in the PVX ELISA. At least three biological replicates were used for each treatment. Values are means ± SD.


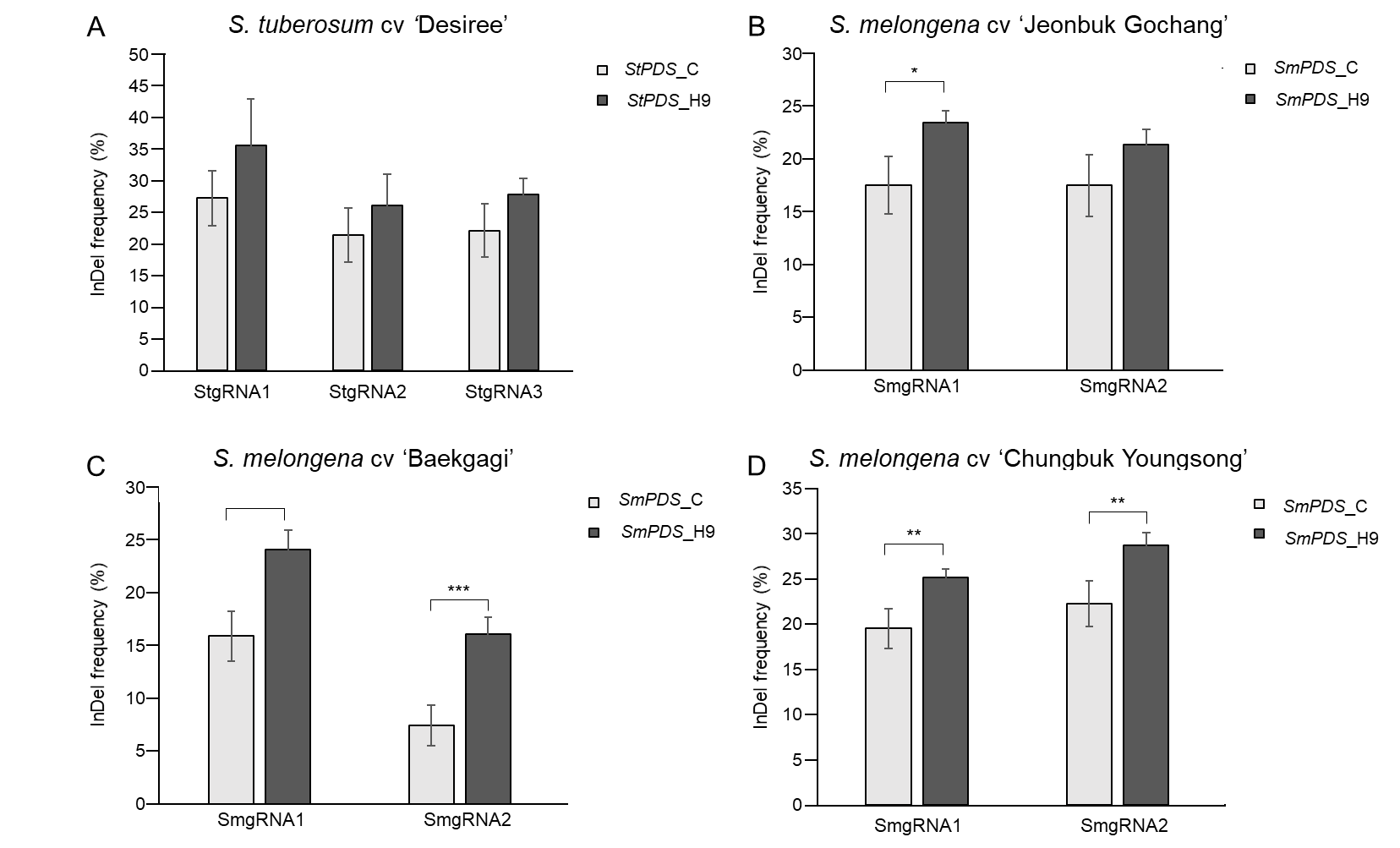


**Fig. S15. Mutation detection and effect of HT on *StPDS* and *SmPDS* editing in PVX-sgRNA -inoculated leaves.**

CAPS analysis was performed on potato and eggplant leaves inoculated with PVX-*sgRNA* at 10 DPI to investigate the editing efficiency and effect of HT in Solanaceous crops. **A** InDel frequency after a 24-h HT in the true leaves of potato cv. ‘Desiree’ inoculated with PVX-*StPDS*. **B** InDel frequency after a 24-h HT in cotyledons of eggplant cv. ‘Jeonbuk Gochang’ inoculated with PVX-*SmPDS*. **C** InDel frequency after a 24-h HT in cotyledons of eggplant cv. ‘Baekgagi’ inoculated with PVX-*SmPDS*. **D** InDel frequency after a 24-h HT in cotyledons of eggplant cv. ‘Chungbuk Youngdong’ inoculated with PVX-*SmPDS*. DNA band intensities in the gel images were analyzed via ImageJ software. At least three biological replicates were used for each treatment. Values are means ± SD.


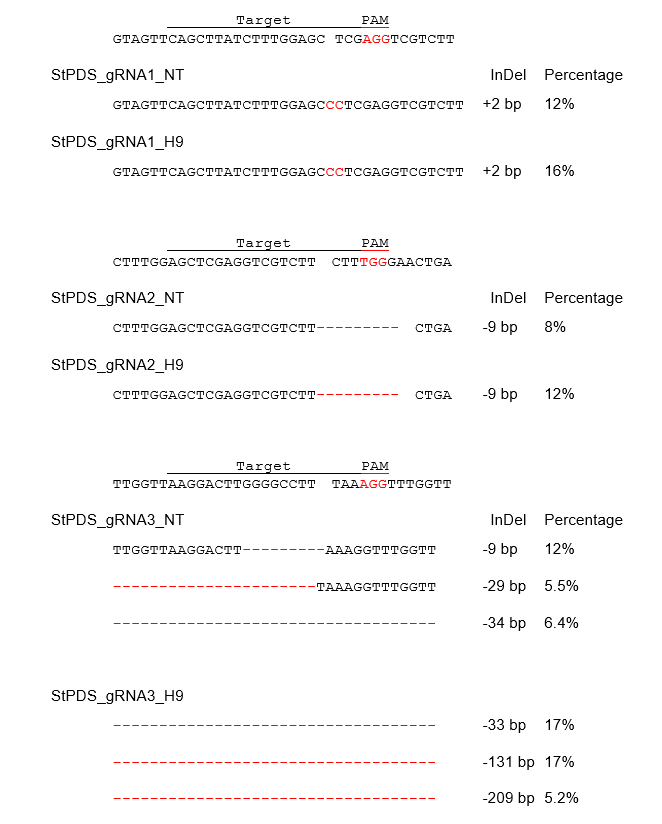


### **Fig. S16. Effects of heat treatment on PVX-mediated GE of *StPDS* in potato analyzed by DECORD tool.**

The *StPDS* target region was amplified from each PVX-StPDS-inoculated leaf, and the InDel frequency was analyzed by the DECORD tool. The types of mutations and the proportion of each mutation are shown on the right. NT, Normal condition (23℃); H9, 37℃ for 24 h starting at 9 DPI.
