# Supplementary material for: Development of virus-induced genome editing methods in Solanaceous crops
Source: Hortic Res. 2023 Nov 17;11(1):uhad233. doi: 10.1093/hr/uhad233 (PMC10782499; doi:10.1093/hr/uhad233)
Supplement: Web_Material_uhad233 [file web_material_uhad233.zip › Fig. S9.pdf]

Target PAM

TTTCTGCTGTTAACTTGAGAGTCCAAGG

Sample	Sequence	InDel	Percentage
NT shoot #11	TTTCTGCTGTTAACTTGAGAGTTCCAAGG	+1 bp	48.3%
NT shoot #64	TTTCTGCTGTTAACT------- CCAAGG	-7 bp	49.7%
NT shoot #66	TTTCTGCTGTTAACTTGAGAGTTCCAAGG	+1 bp	49.2%
NT shoot #67	TTTCTGCTGTTAACTTGAGAGTTCCAAGG	+1 bp	51.1%
HT shoot #13	TTTCTGCTGTTAACTTGAGAGT -CAAGG	-1 bp	50.7%
	TTTCTGCTGTTAACTTGAGAGTTCCAAGG	+1 bp	23.9%
HT shoot #14	TTTC------------------ -CAAGG	-19 bp	51.0%
	TTTCTGCTGTTAACTTGAGAGTTCCAAGG	+1 bp	49.0%
HT shoot #15	TTTCTGCTGTTAACTT------ CCAAGG	-6 bp	24.6%
	TTTCTGCTGTTAACTTGAGAGTTCCAAGG	+1 bp	24.4%
	TTTCTGCTGTTAACTTGAGAGTAAGTCCAAGG	+4 bp	14.6%
	TTTCTGCTGTTAACTTGAGAGTAAATCCAAGG	+4 bp	9.4%
HT shoot #19	TTTCTGCTGTTAACTTGAGAGTTCCAAGG	+1 bp	49.2%
	TTTCTGCTGTTAACTTGA-- TTCCAAGG	-3/+1 bp	38.6%
	TTTCTGCTGTTAACTTGA-- GTCCAAGG	-3/+1 bp	12.2%
HT shoot #20	TTTCTGCTGTTAACTTGAGAGTTCCAAGG	+1 bp	70.5%
HT shoot #21	TTTCTGCTGTTAACTTGAGAGTTCCAAGG	+1 bp	50.0%
HT shoot #22	TTTCTGCTGTTAACTTGAGAGTTCCAAGG	+1 bp	48.0%
HT shoot #23	TTTCTGCTGTTAACTTGAGAGTTCCAAGG	+1 bp	53.6%
HT shoot #26	TTTCTGCTGTTAACTTGAGAGTTCCAAGG	+1 bp	40.2%
HT shoot #27	TTTCTGCTGTTAACTTGAGAGTTCCAAGG	+1 bp	46.2%
HT shoot #31	TTTCTGCTGTTAACTTGAGAGTTCCAAGG	+1 bp	23.7%
HT shoot #38	TTTCTGCTGTTAACTTGAGAGTTCCAAGG	+1 bp	44.8%
HT shoot #39	TTTCTGCTGTTAACTTGAGAGTTCCAAGG	+1 bp	55.0%
HT shoot #40	TTTCTGCTGTTAACTTGAGAGTTCCAAGG	+1 bp	51.8%
HT shoot #43	TTTCTGCTGTTAACT------- CCAAGG	-7 bp	54.9%
HT shoot #50	TTTCTGCTGTTAACTTGAGAGTTCCAAGG	+1 bp	49.6%
HT shoot #51	TTTCTGCTGTTAACTTGAGAGTTCCAAGG	+1 bp	59.8%
HT shoot #53	TTTCTGCTGTTAACTT------ CCAAGG	-6 bp	53.6%
	TTTCTGCTGTTAACTTGAGA-- CCAAGG	-2 bp	46.4%
HT shoot #58	TTTCTGCTGTTAACTTGAGAGTTCCAAGG	+1 bp	27.0%
	TTTCTGCTGTTAAC-------- CCAAGG	-8 bp	18.5%
HT shoot #59	TTTCTGCTGTTAACTTGAGAGTTCCAAGG	+1 bp	42.2%
	---------------------- CCAAGG	-3 bp	5.4%
HT shoot #60	TTTCTGCTGTTAACTTGAGAGTTCCAAGG	+1 bp	55.1%
	TTTCTGCTGTTAACTTGAG--- CCAAGG	-3 bp	44.9%
HT shoot #64	TTTCTGCTGTTAACT------- CCAAGG	-7 bp	49.7%
HT shoot #66	TTTCTGCTGTTAACTTGAGAGTTCCAAGG	+1 bp	49.2%
HT shoot #67	TTTCTGCTGTTAACTTGAGAGTTCCAAGG	+1 bp	51.1%
