# Supplementary material for: Development of virus-induced genome editing methods in Solanaceous crops
Source: Hortic Res. 2023 Nov 17;11(1):uhad233. doi: 10.1093/hr/uhad233 (PMC10782499; doi:10.1093/hr/uhad233)
Supplement: Web_Material_uhad233 [file web_material_uhad233.zip › Fig.-S1.PDF]

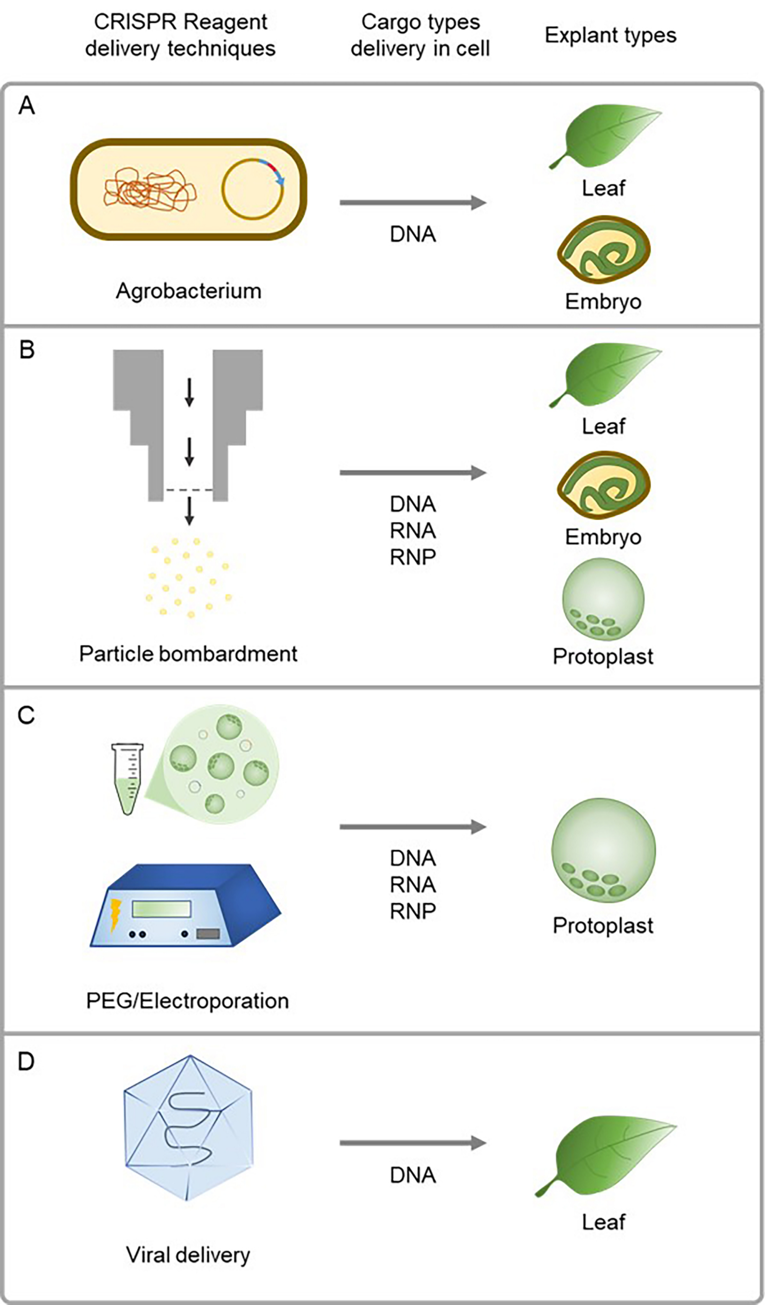

CRISPR Reagent delivery techniques
Cargo types delivery in cell
Explant types
A
DNA
Leaf
Embryo
Agrobacterium
B
DNA
RNA
RNP
Leaf
Embryo
Protoplast
Particle bombardment
C
DNA
RNA
RNP
Protoplast
PEG/Electroporation
D
DNA
Leaf
Viral delivery
