# Supplementary material for: Development of virus-induced genome editing methods in Solanaceous crops
Source: Hortic Res. 2023 Nov 17;11(1):uhad233. doi: 10.1093/hr/uhad233 (PMC10782499; doi:10.1093/hr/uhad233)

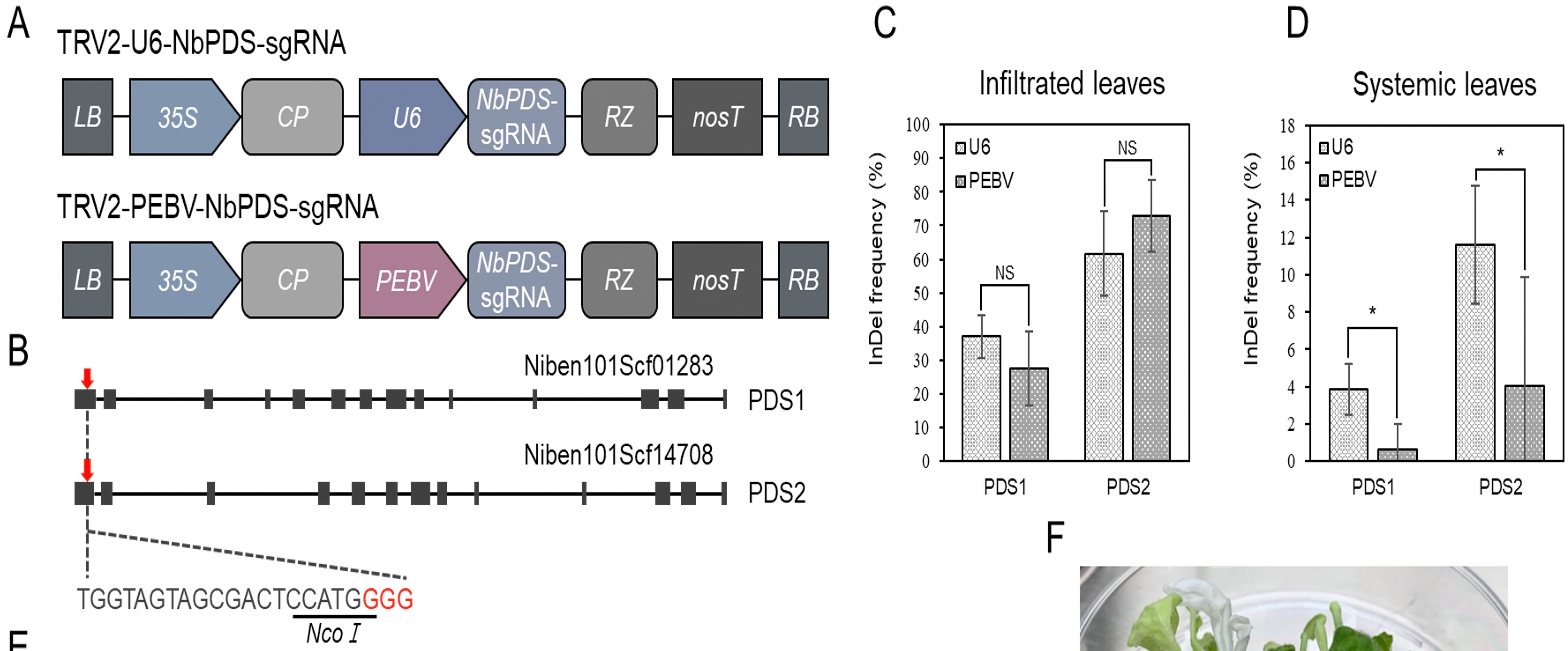

E

No. of shoots	U6		PEBV	
	PDS1	PDS2	PDS1	PDS2
Wild-type	2	1	11	7
Monoallelic mutnat	10	10	5	9
Biallelic mutant	0	1	0	0
Murant ratio	83.3%	91.7%	31.3%	56.3%

F

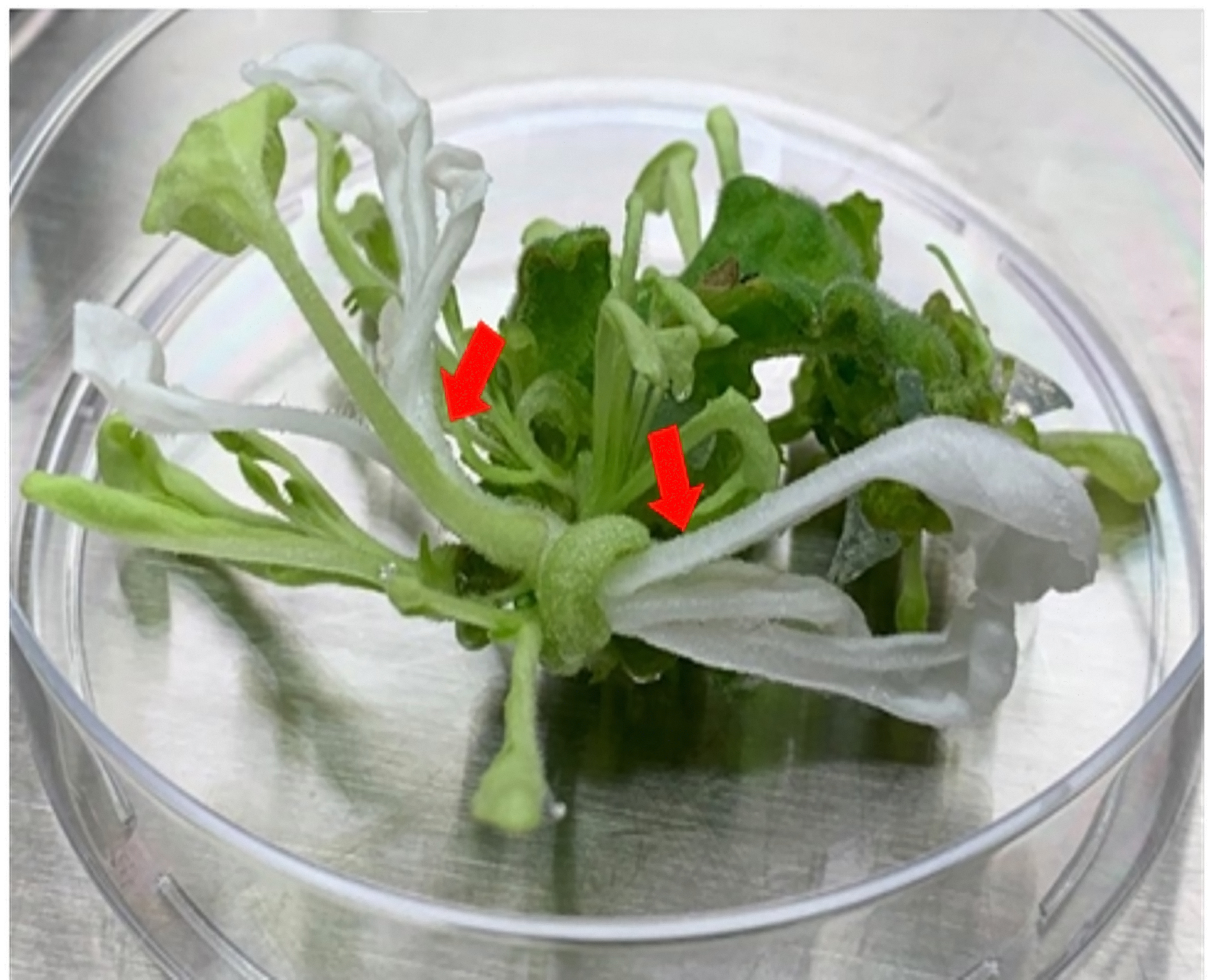

Supplement: Web_Material_uhad233 [file web_material_uhad233.zip › Fig.-S4.PDF]
