# Supplementary figures and images for: Development of virus-induced genome editing methods in Solanaceous crops
Source: Hortic Res. 2023 Nov 17;11(1):uhad233. doi: 10.1093/hr/uhad233 (PMC10782499; doi:10.1093/hr/uhad233)

## Transformation GE method (5 ~ 7 months)

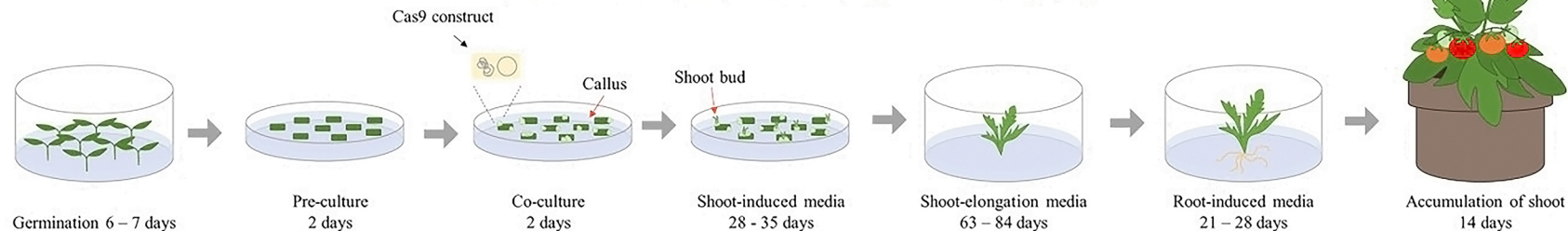

## VIGE method (3 ~ 5 months)

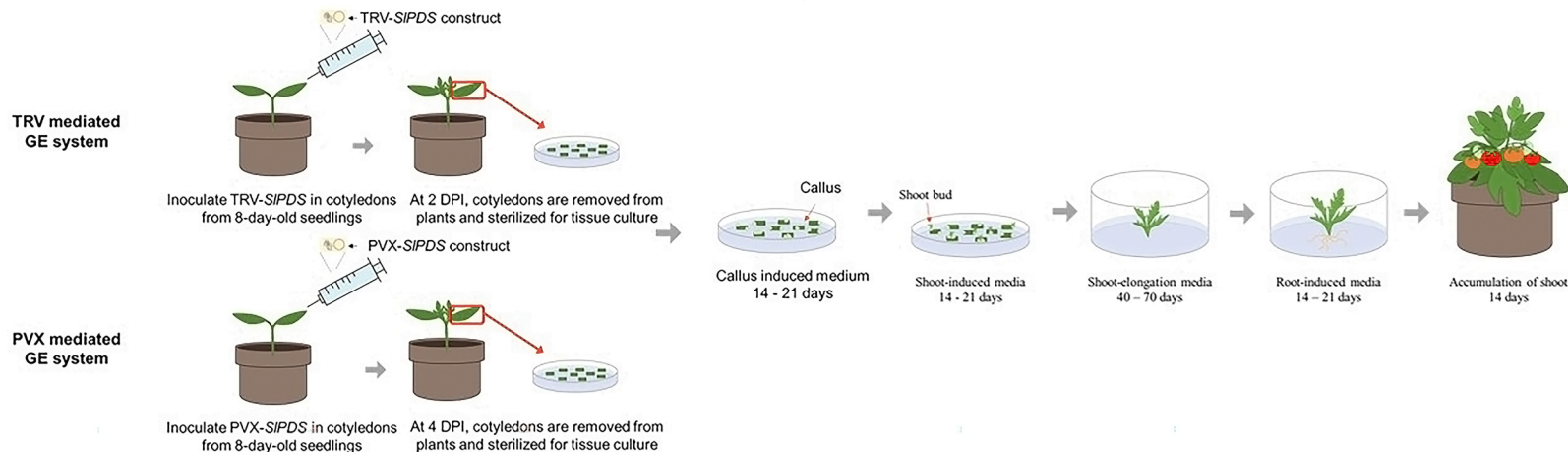

Supplement: Web_Material_uhad233 [file web_material_uhad233.zip › Fig. S2.pdf]

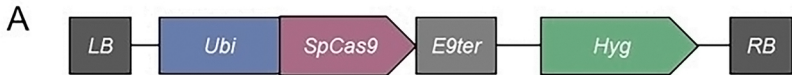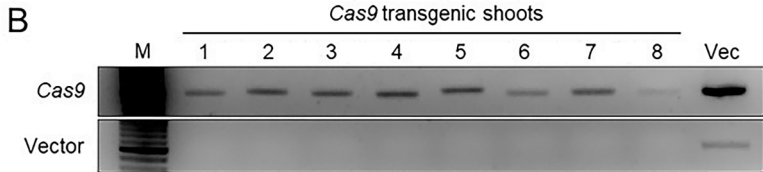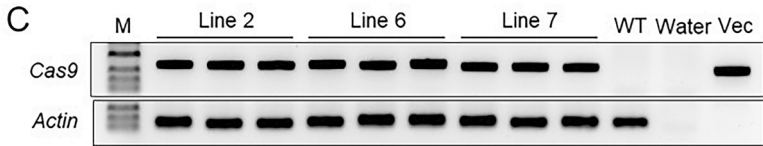

Supplement: Web_Material_uhad233 [file web_material_uhad233.zip › Fig. S3.pdf]

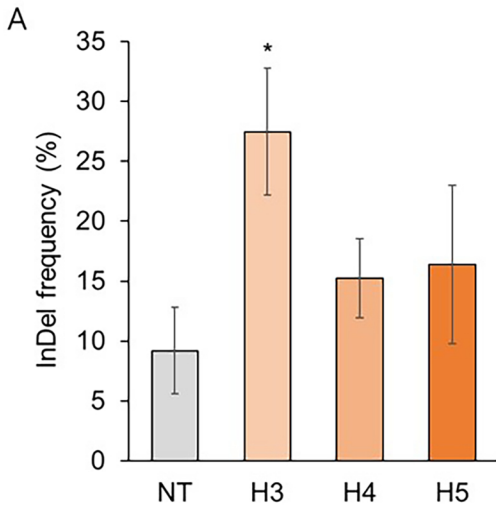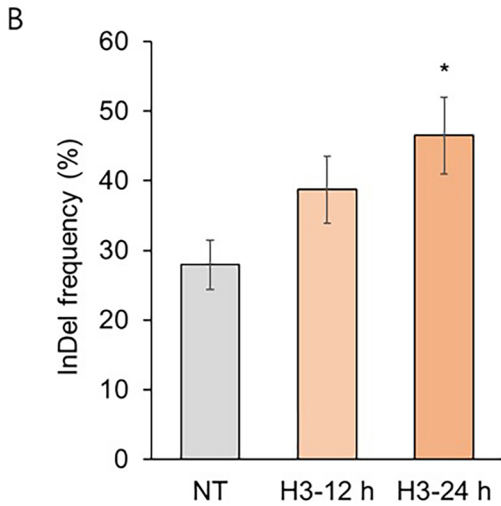

Supplement: Web_Material_uhad233 [file web_material_uhad233.zip › Fig. S5.pdf]

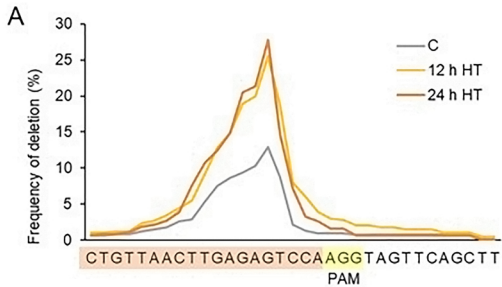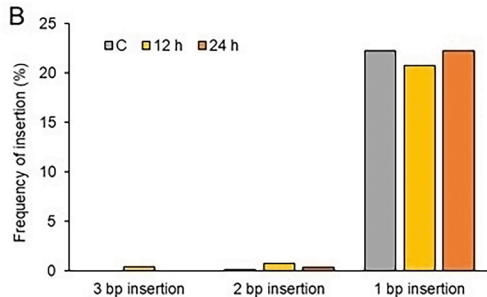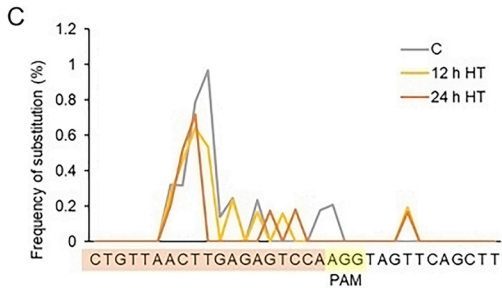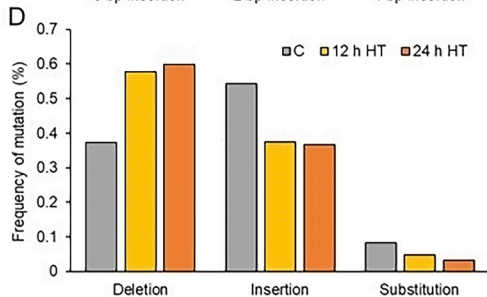

Supplement: Web_Material_uhad233 [file web_material_uhad233.zip › Fig. S6.pdf]

A

TRV2-SIPDS-sgRNA

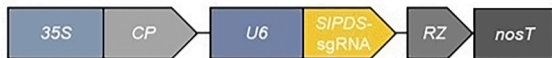

TRV2-SIPDS-sgRNA-FT

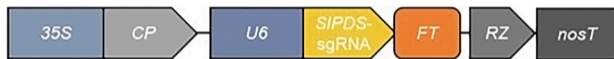

TRV2-SIPDS-sgRNA-tRNA

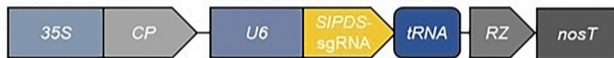

B

Solyc03g123760.3.1

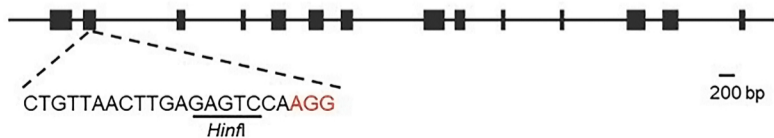

Supplement: Web_Material_uhad233 [file web_material_uhad233.zip › Fig. S7.pdf]

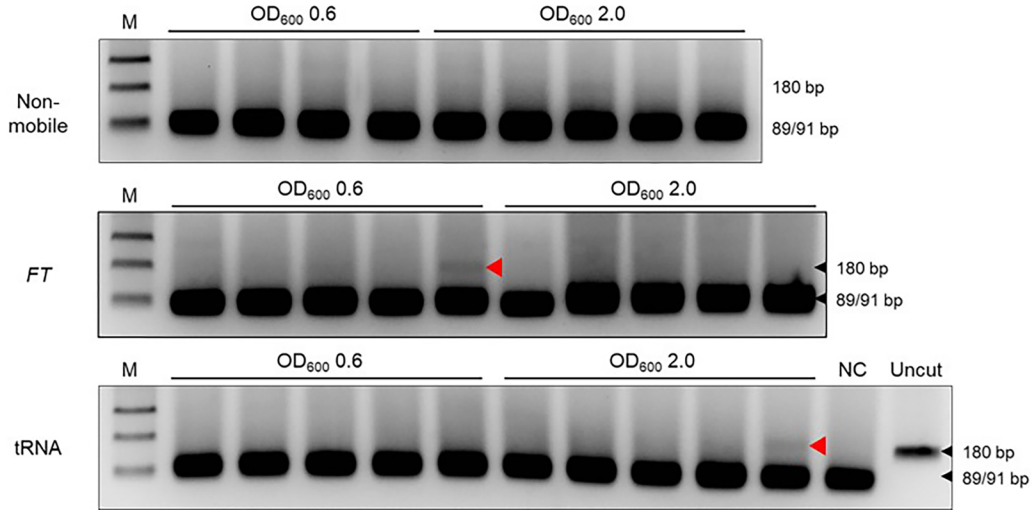

Supplement: Web_Material_uhad233 [file web_material_uhad233.zip › Fig. S8.pdf]

M

*SIPDS* E<sub>1</sub> mutants

PC

*TRV CP*

*SlActin*

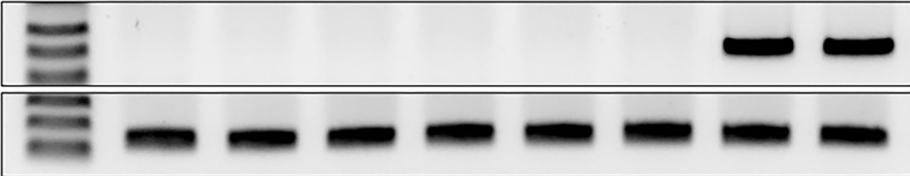

Supplement: Web_Material_uhad233 [file web_material_uhad233.zip › Fig. S10.pdf]

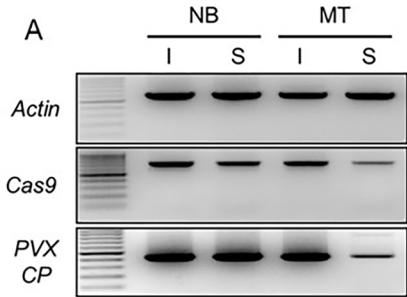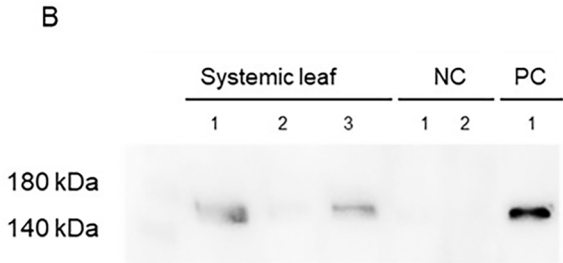

Supplement: Web_Material_uhad233 [file web_material_uhad233.zip › Fig. S11.pdf]

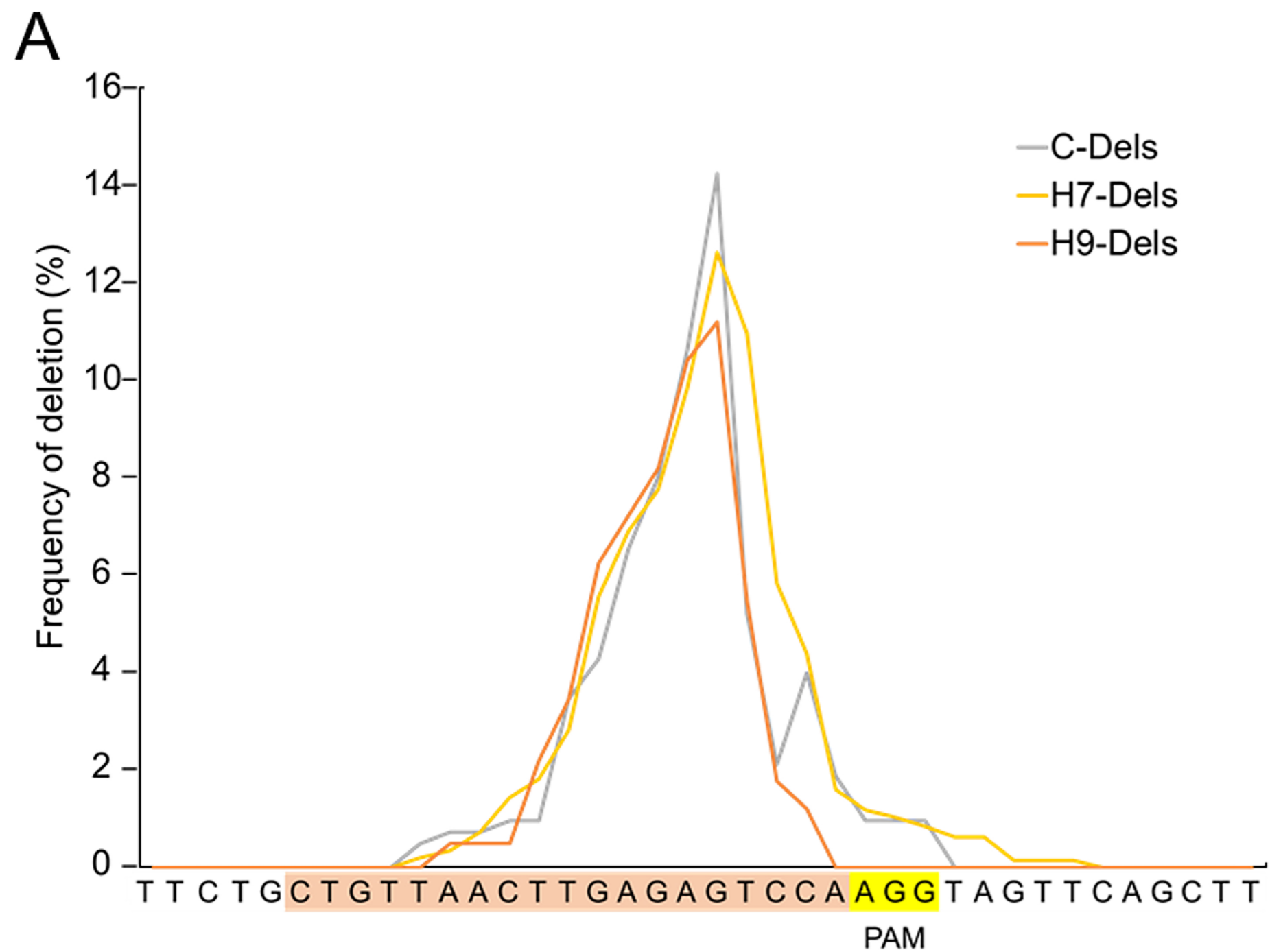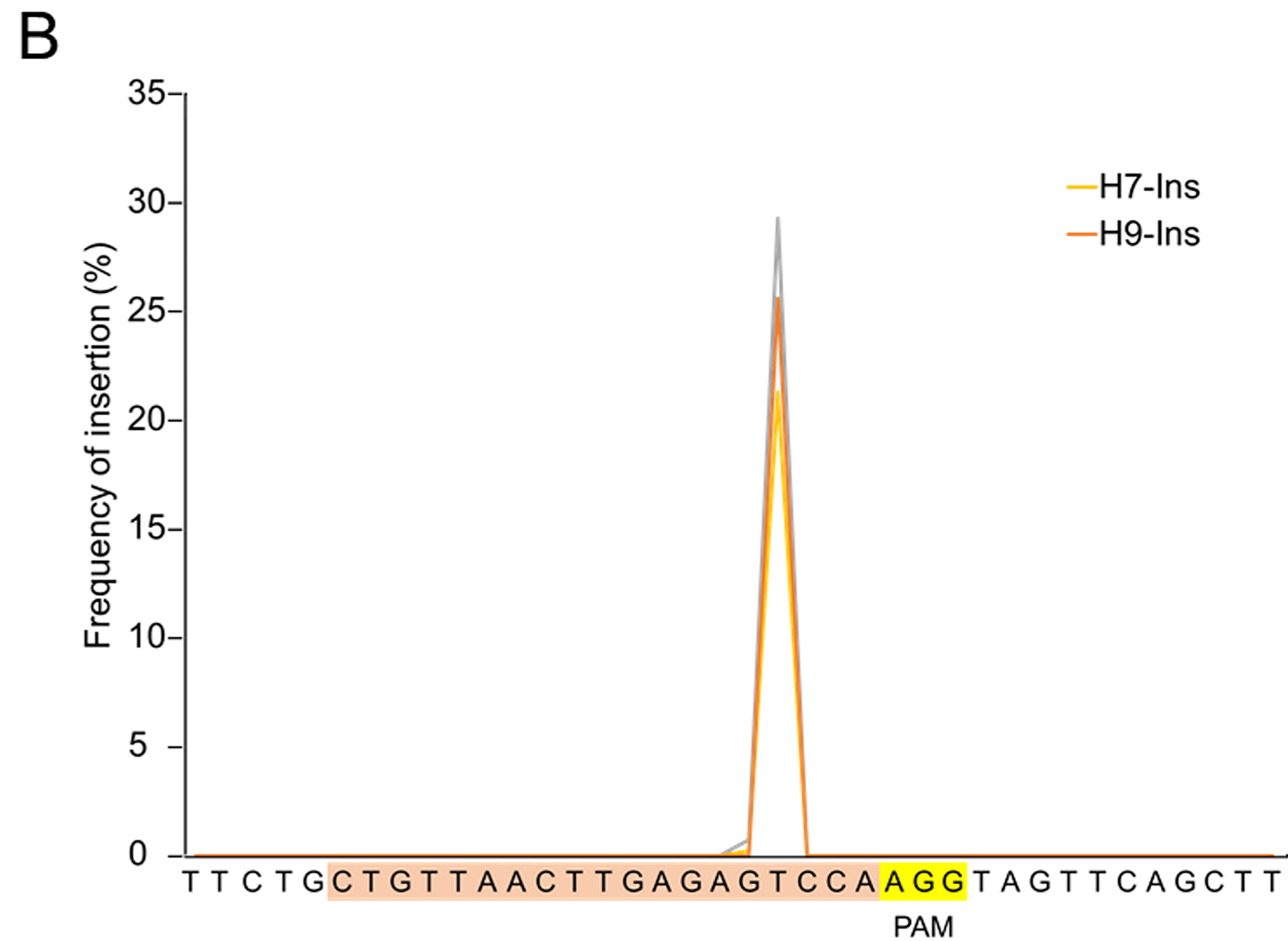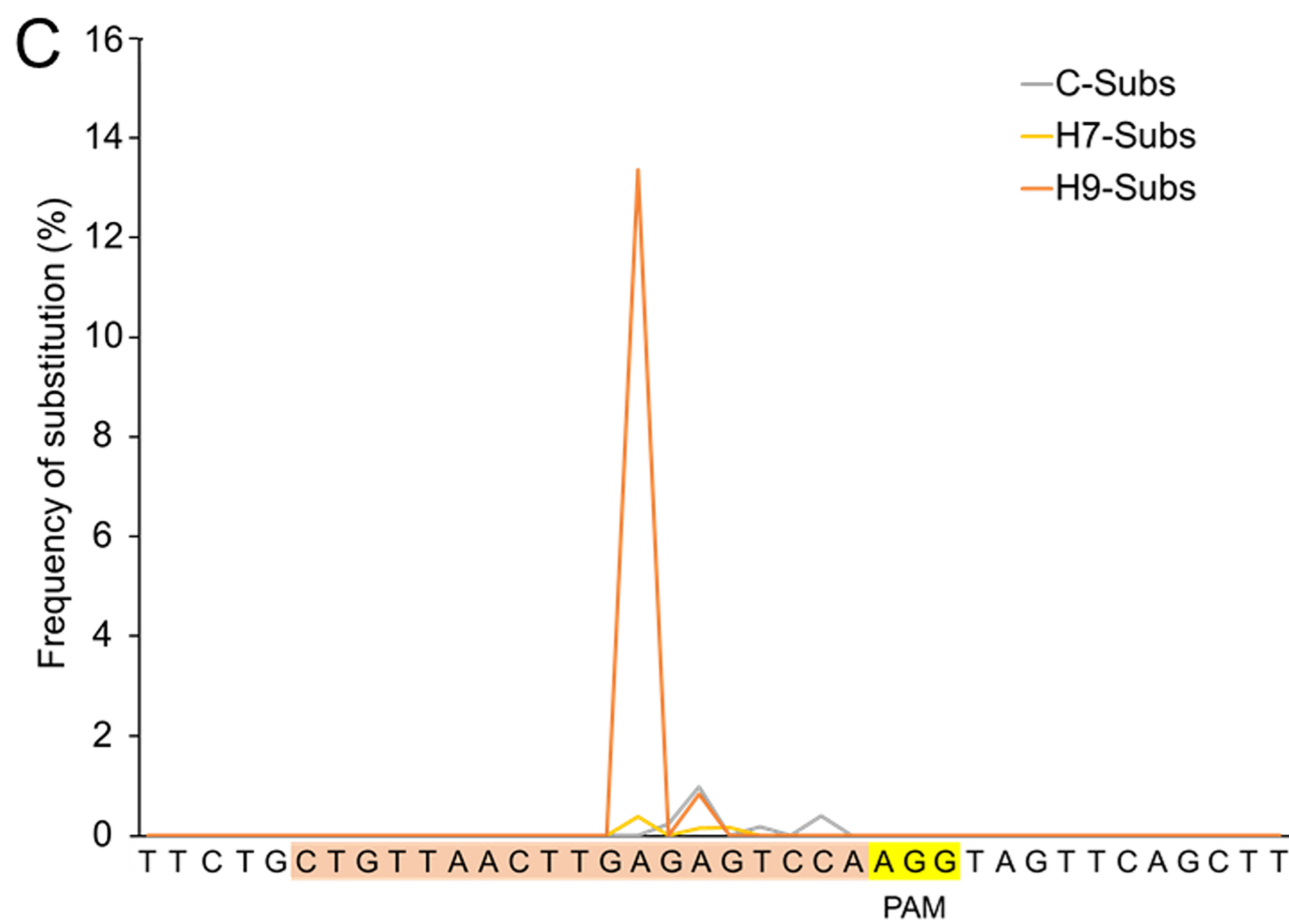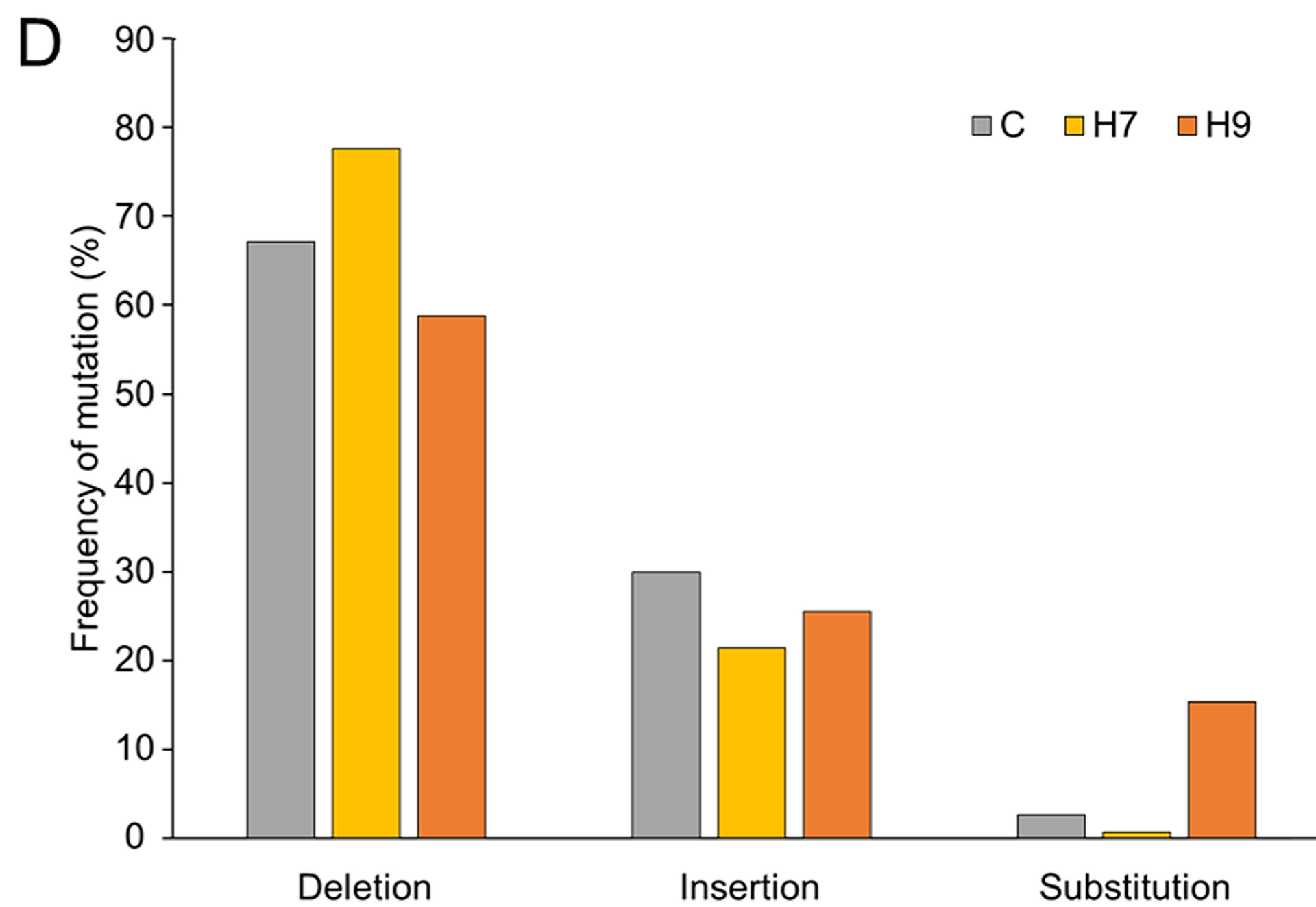

Supplement: Web_Material_uhad233 [file web_material_uhad233.zip › Fig. S13.pdf]

A

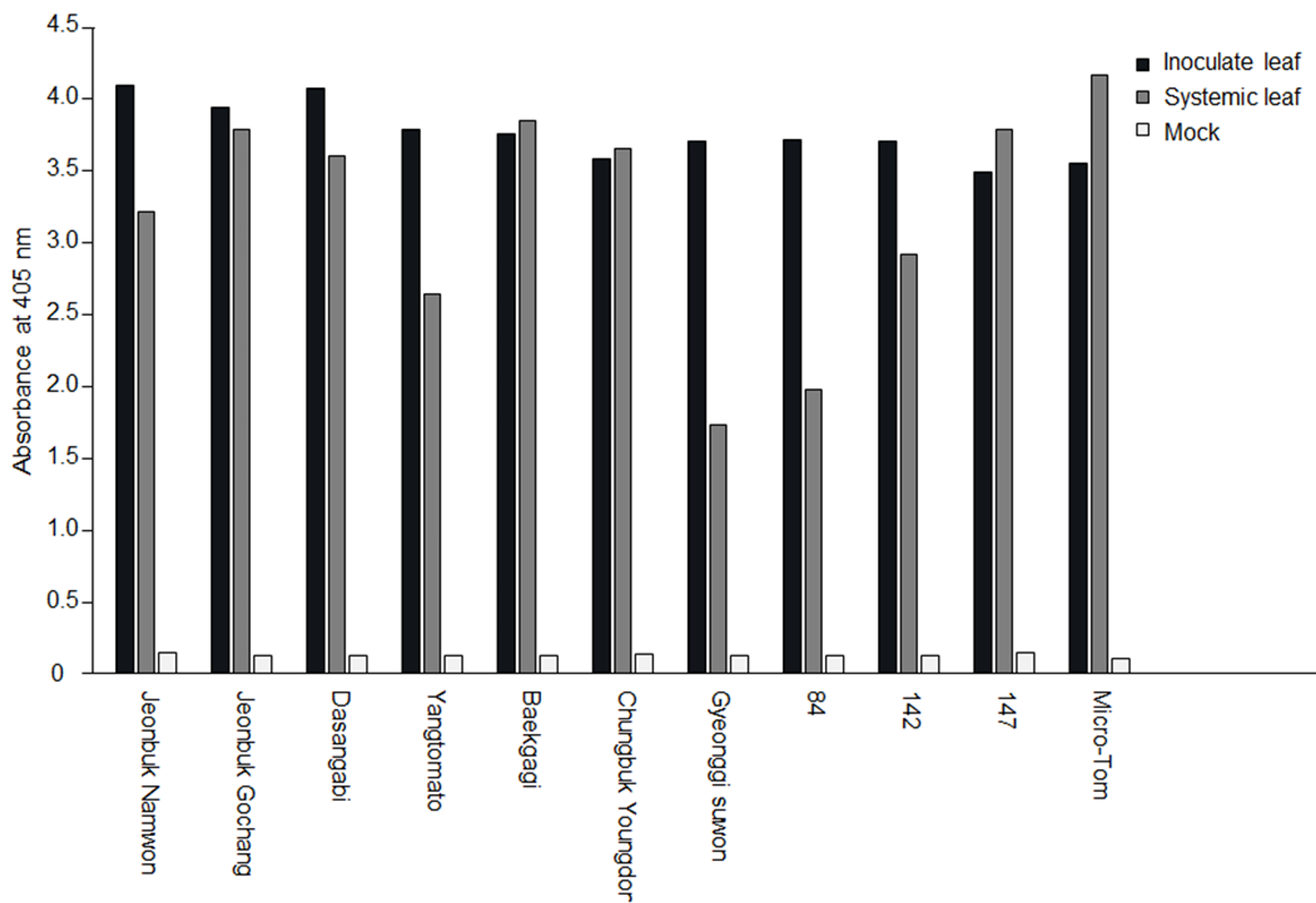

B

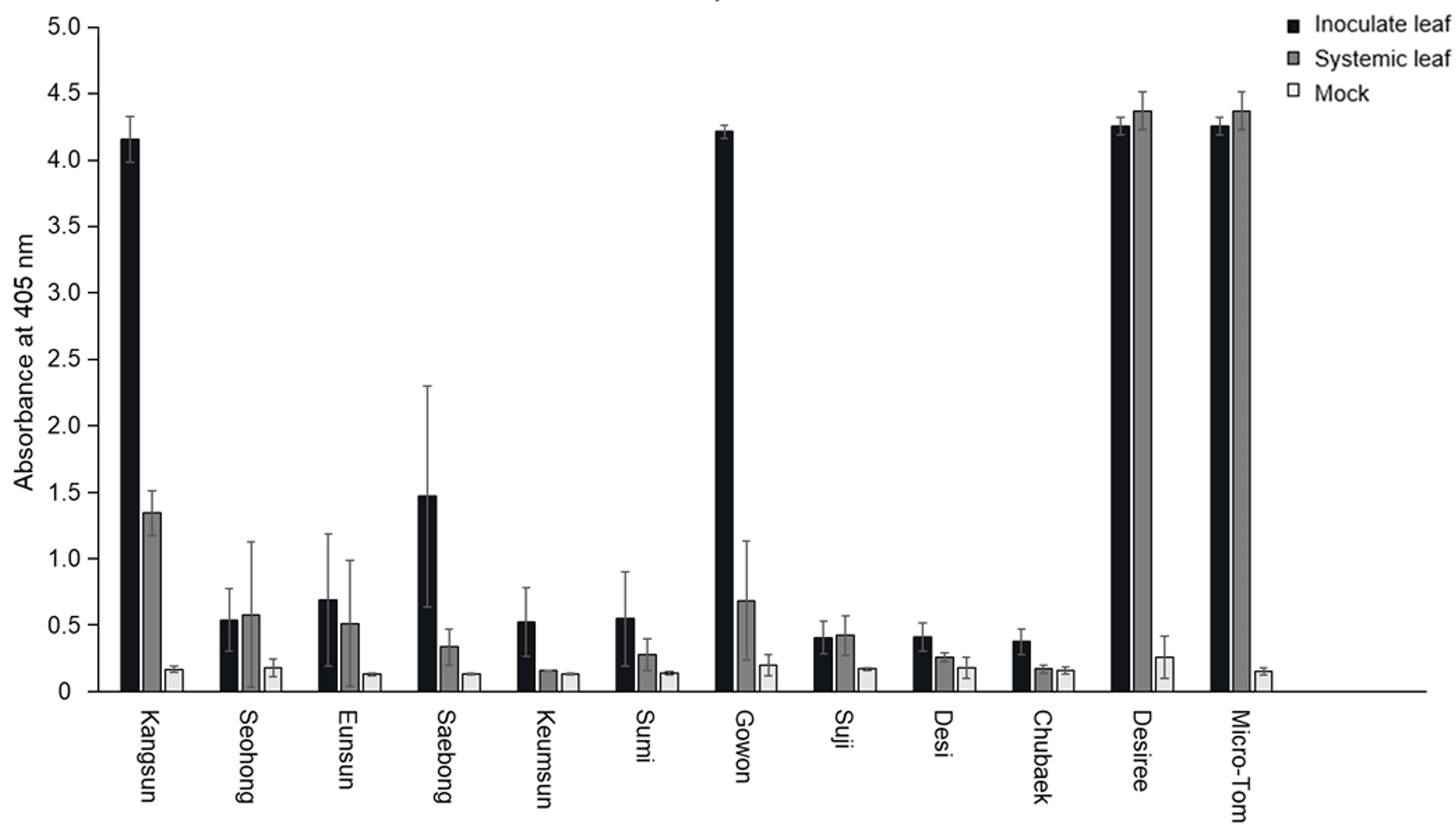

Supplement: Web_Material_uhad233 [file web_material_uhad233.zip › Fig. S14.pdf]

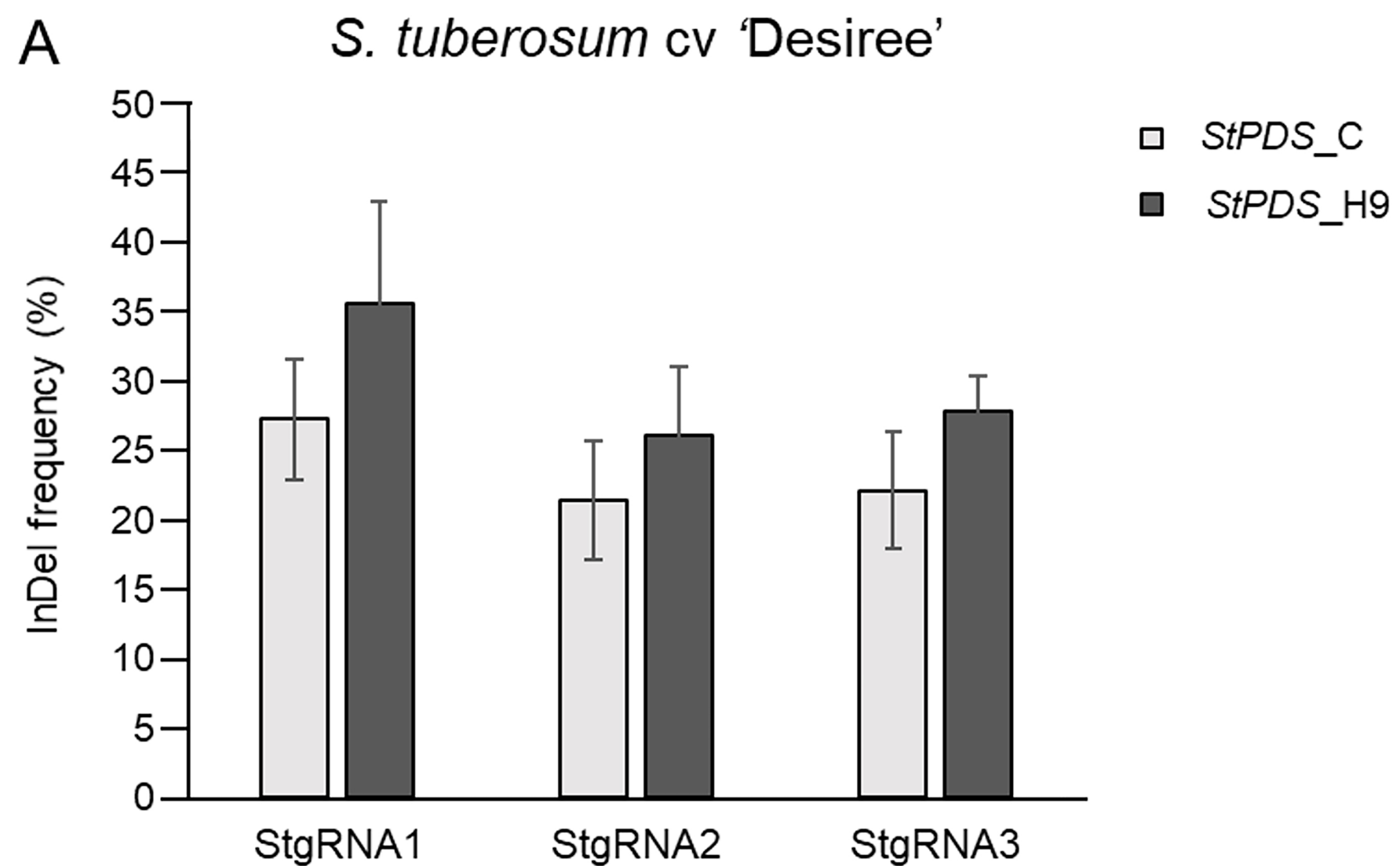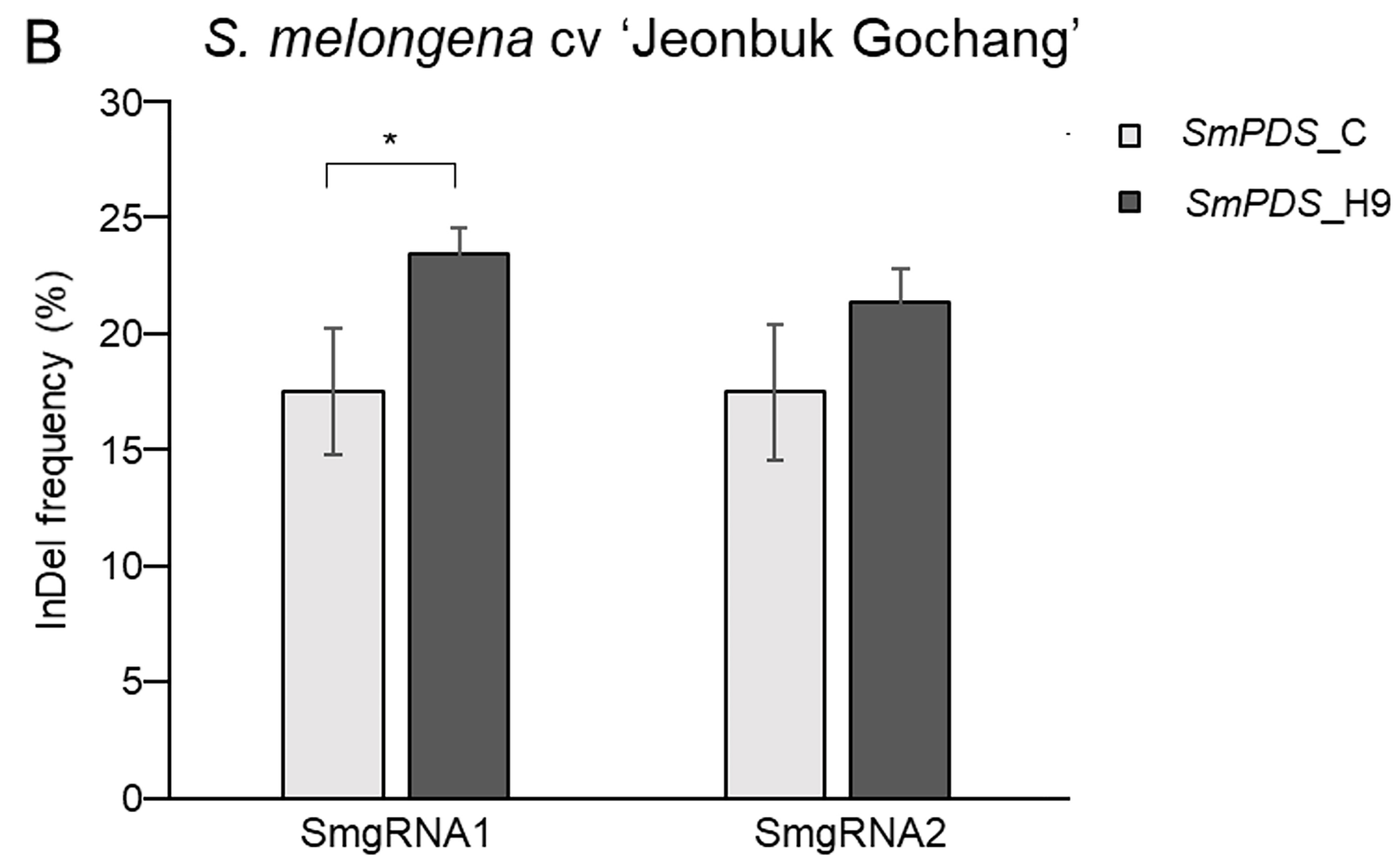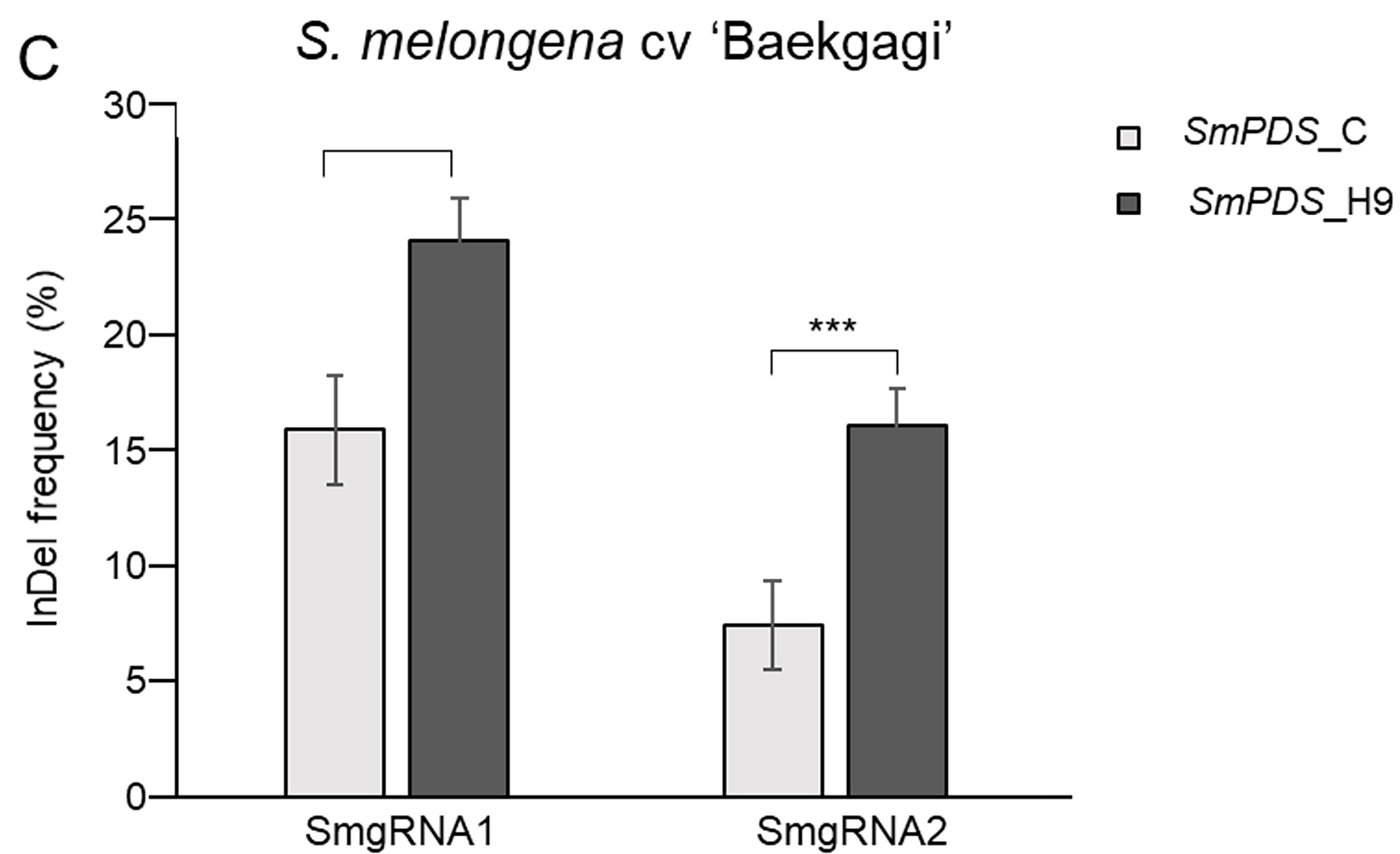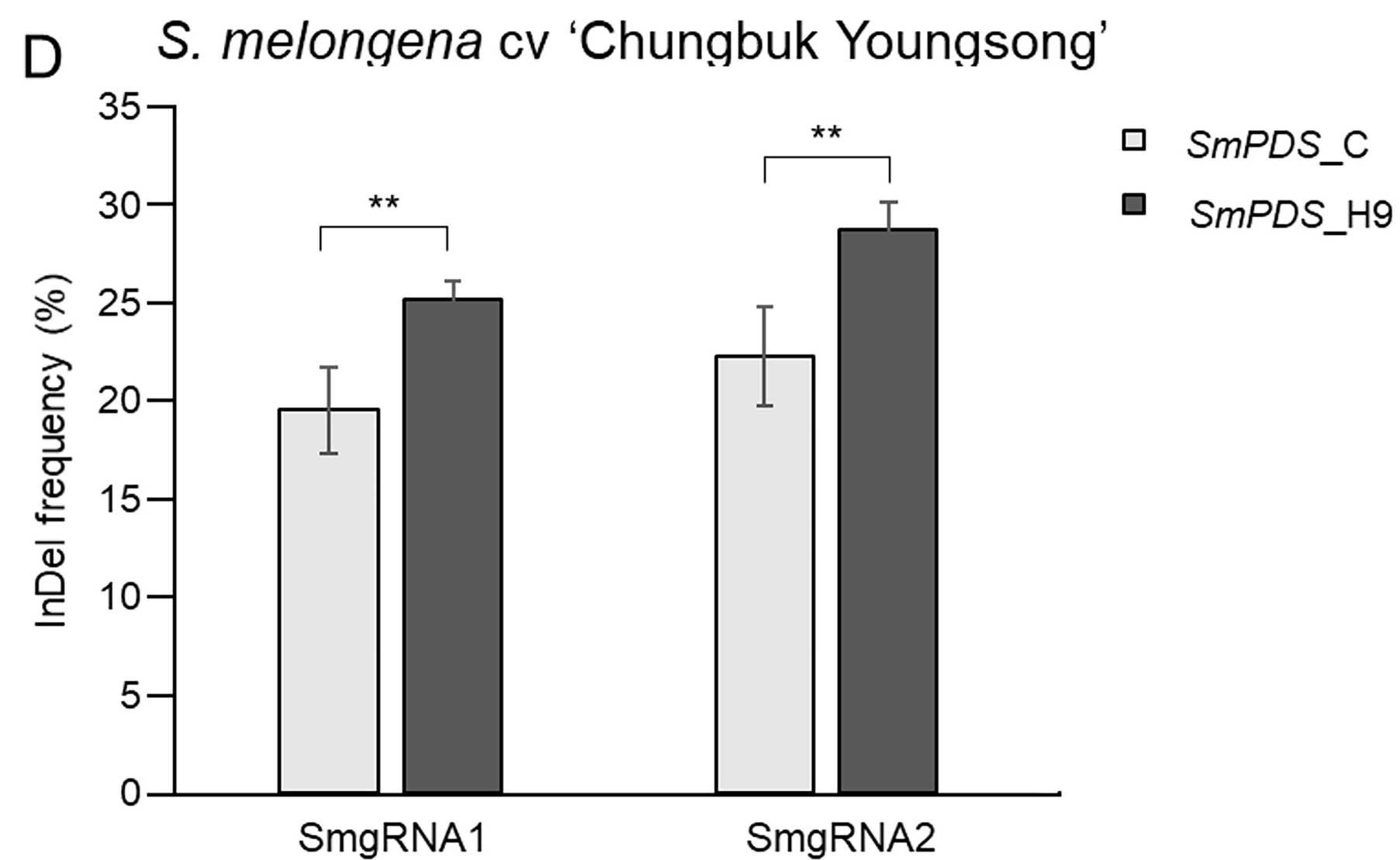

Supplement: Web_Material_uhad233 [file web_material_uhad233.zip › Fig. S15.pdf]

**A**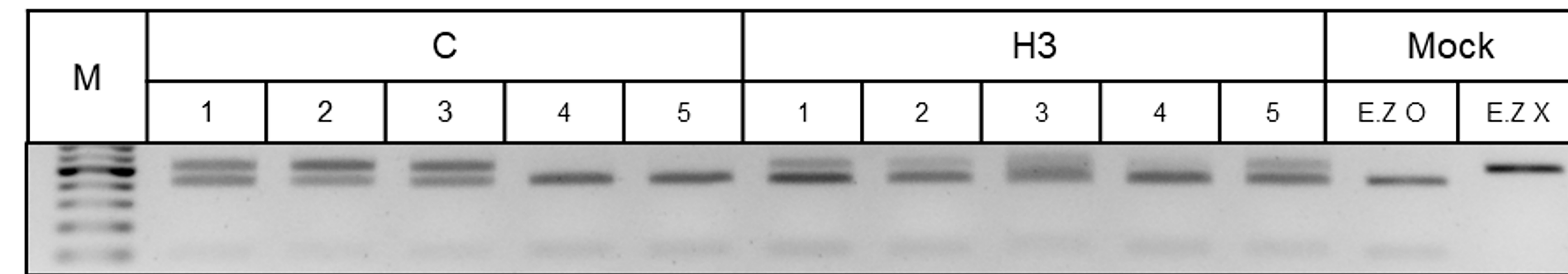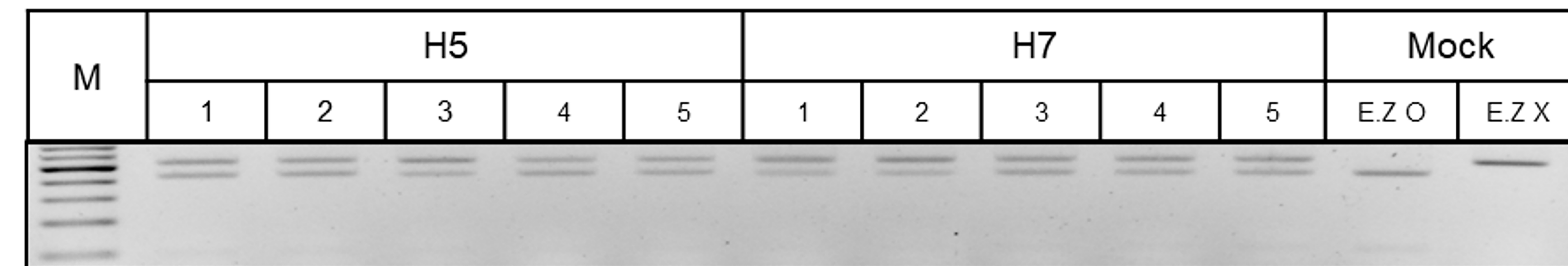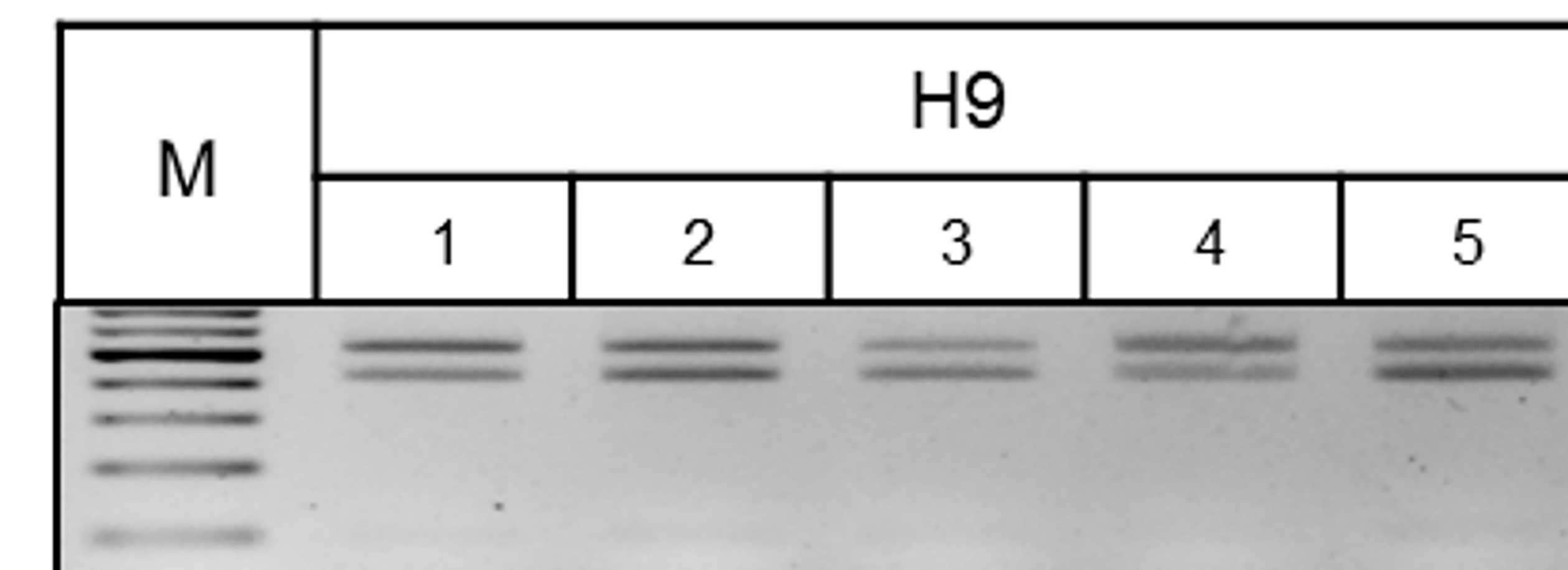**B**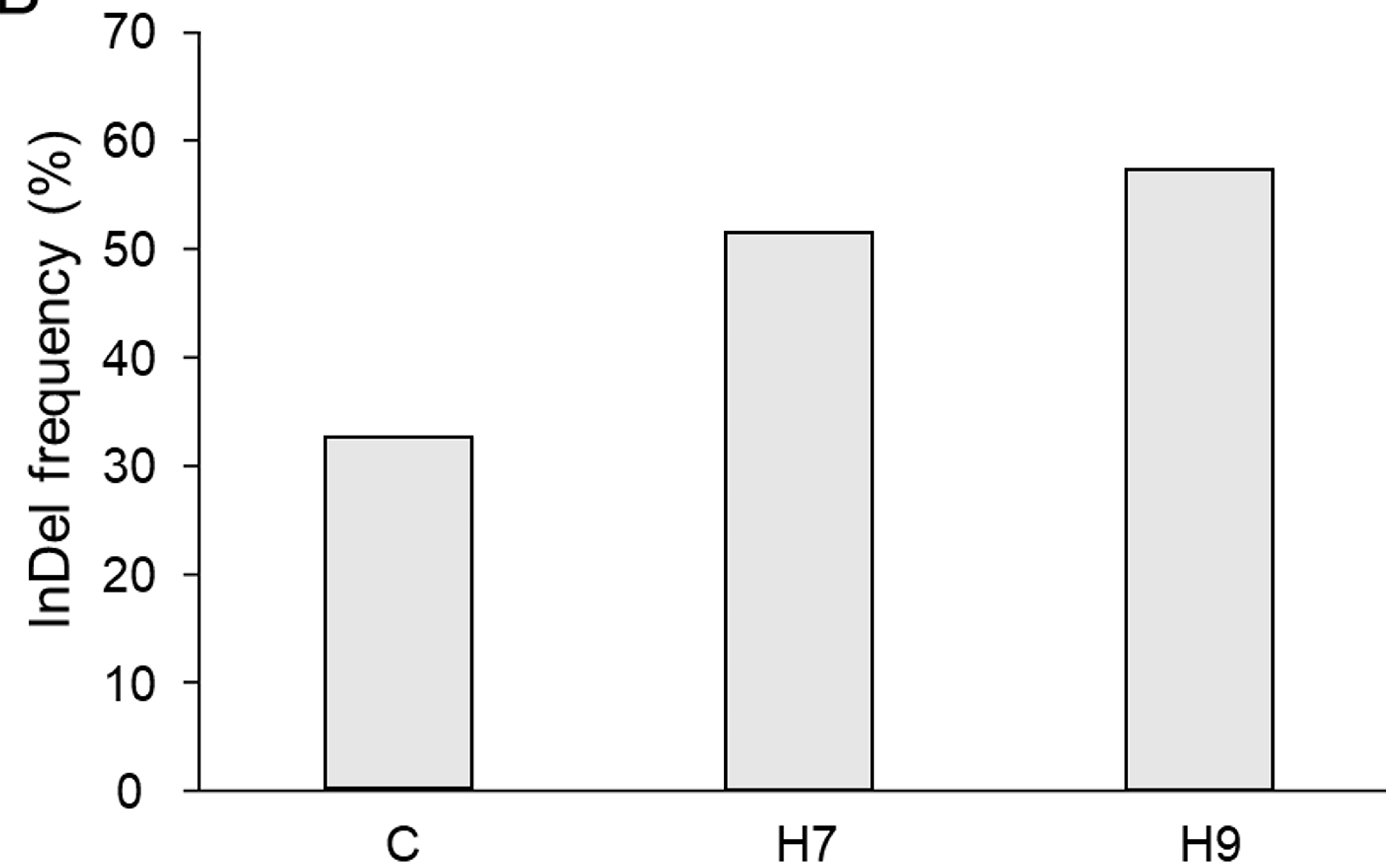**C**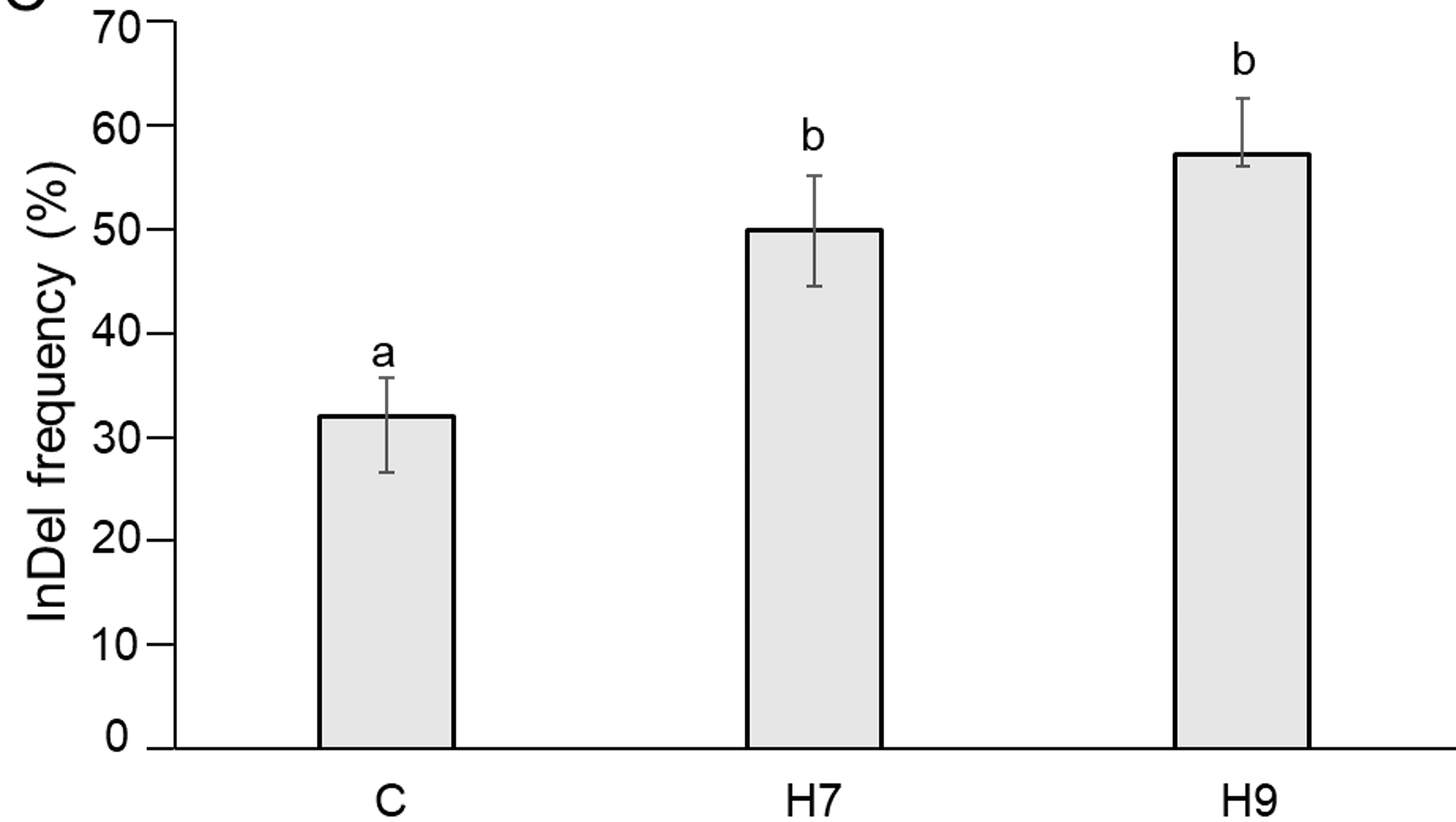

Supplement: Web_Material_uhad233 [file web_material_uhad233.zip › FIg.-S12.PDF]
